# The Optically Guided and Pre-assembled Implantation Cranial Window Reveals Cortical Spatial Representations during Navigation

**DOI:** 10.34133/research.1072

**Published:** 2026-01-15

**Authors:** Weihao Zhao, Lin Gao, Yu Wu, Guihua Xiao, Angran Li, Mingrui Wang, Hongyu Liu, Jinyuan Yang, Dongyue Jin, Yuanlong Zhang, Yongyi Yuan, Pu Dai

**Affiliations:** ^1^ Senior Department of Otolaryngology Head and Neck Surgery, The 6th Medical Center of Chinese PLA General Hospital, Chinese PLA Medical School, Beijing 100853, China.; ^2^ State Key Laboratory of Hearing and Balance Science, Beijing 100853, China.; ^3^ National Clinical Research Center for Otolaryngologic Diseases, Beijing 100853, China.; ^4^ Key Laboratory of Hearing Science, Ministry of Education, Beijing 100853, China.; ^5^ Beijing Key Laboratory of Hearing Impairment Prevention and Treatment, Beijing 100853, China.; ^6^ Department of Otolaryngology, General Hospital of the Tibet Military Region, Lhasa 850000, China.; ^7^ Department of Automation, Tsinghua University, Beijing 100084, China.; ^8^ School of Information Science and Technology, Fudan University, Shanghai 200433, China.; ^9^ Department of Neurosurgery, Hainan Hospital of Chinese PLA General Hospital, Sanya 572010, China.; ^10^ School of Life Sciences, Tsinghua University, Beijing 100084, China.

## Abstract

Head-mounted miniaturized microscopes have provided new capabilities for neuroscience by enabling neural imaging in freely behaving animals. The long-term application of these microscopes, however, is contingent upon specific and reliable cranial window designs. Here, we introduce optically guided and pre-assembled implantation (OGPI), a standardized cranial window technique designed for head-mounted miniaturized microscope imaging. OGPI employs a cost-effective, minimalist design and offers robust compatibility with miniaturized microscopes. This integrated method ensures precise implantation and supports chronic large-scale neural imaging in freely behaving animals for periods exceeding 8 months. The OGPI method is adaptable, supporting both semiautomated operation for enhanced throughput and manual operation for standard laboratory settings. Through behavioral assessments, we further demonstrate that animals with OGPI cranial windows exhibit preserved locomotor and spatial cognitive abilities. Leveraging this chronic window, we performed large-scale cortical imaging in mice engaged in a Y-maze navigation task. We found that the neurons’ tuning position, path, and acceleration were distributed in a “salt-and-pepper” pattern across multiple cortices. A subpopulation of neurons exhibiting conjunctive tuning to both spatial information and linear acceleration was identified, suggesting that the acceleration-tuned neurons required for the generation and updating of the spatial signal exist in the cortex. Further, population-level analyses of spatial representation in the cortex were conducted. A decoder and a classifier based on cortical activity accurately predicted the animal’s position and path. Altogether, our results establish OGPI as an enabling platform and a key methodological advancement for chronic imaging in freely behaving animals and reveal a widespread representation of spatial information in the cortex.

## Introduction

Understanding how the mammalian brain encodes, processes, and stores sensory information demands the ability to monitor the coordinated activity of entire neural networks at cellular resolution, during natural behaviors [1]. Research in systems neuroscience has revealed that neurons do not function in isolation—instead, neural representation emerges from the collective dynamics of neuronal populations [2–4]. Hence, to unravel brain computations, we need tools to record from all or most neurons in a functional circuit simultaneously, over time scales that capture both rapid neural dynamics and long-term changes [5,6]. Achieving this goal remains challenging: experiments must allow animals to behave naturally and employ their full behavioral repertoire, as behavior profoundly shapes neural activity [4,7]. At the same time, neural recording techniques must remain stable over days to months to trace how circuit activity and synaptic “wiring” evolve during learning and memory formation [8].

Optical interrogation with genetically encoded calcium indicators has substantially advanced systems neuroscience by enabling chronic, cell-type-specific recordings from thousands of neurons without the inflammatory scarring inherent to penetrating electrodes [6,9,10]. When combined with head-mounted 1- and 2-photon microscopes that now weigh <4 g, researchers can dissect how distributed circuits orchestrate decision-making and learning under freely behaving conditions [11,12]. However, achieving single-cell resolution across multiple brain regions in freely behaving mice remains challenging due to both optical throughput constraints within limited cranial real estate and the difficulty of maintaining long-term, stable imaging windows.

Chronic large cranial windows necessitate replacing the skull with a transparent substrate—a technically demanding procedure with several major hurdles [13]. First, the risk of fatal hemorrhage is high, especially when the procedure involves major dural sinuses [14,15]; as a result, many cranial window designs intentionally avoid these regions [14–17]. Second, imaging durability is often compromised over time by infection or connective tissue hyperplasia. Preserving meningeal integrity, periodically clearing hyperplasia [13], and employing antifibrotic materials [14] can help maintain optical clarity. Third, the stability of the cranial window is further challenged by the mechanical stress imposed by the head-mounted miniaturized microscopes during animal movement. In addressing these challenges, precise control over window size and geometry is required. Furthermore, due to the technical difficulties, it remains unclear whether large cranial windows alter sensory perception or learning, highlighting the need to evaluate the impact of a large cranial window on neural and behavioral outcomes.

To implant the cranial window, 2 strategies are currently used: most labs depend on manual operation (marker dotting and surgical feel) that offer limited precision [13,18], while a minority develop complex robotic systems [16,19]. Here, we introduce the optically guided and pre-assembled implantation (OGPI) method, which enables semiautomated, high-throughput implantation with high precision. The OGPI approach is designed to ensure accurate implantation, such that the glass component of the glass–metal unit is embedded within the craniotomy margin and the metal component is securely seated on the cranial rim, thereby enhancing mechanical stability and reducing the risks of infection and connective tissue hyperplasia. To evaluate the chronic stability of OGPI implants and their functional impact, we further performed qualitative and quantitative analyses of long-term neural imaging data and behavioral metrics, including general locomotion and spatial cognition, in mice equipped with OGPI cranial windows.

Using the OGPI platform, we achieved chronic, large-scale neural recordings during freely moving behavior, revealing that spatial representation extends beyond the hippocampal–entorhinal system [20,21] to involve broader cortical regions. Key evidence includes the following: We observed spatially tuned neurons in a “salt-and-pepper” pattern across multiple cortical regions during a navigation task in a Y-maze; we identified neurons exhibiting conjunctive tuning to both spatial information and linear acceleration. We successfully decoded the direction of transitions between 2 regions of the Y-maze with an accuracy of 77.33% and decoded the animal’s position within the Y-maze with an accuracy of 84.98%. In addition, we performed neural manifold analyses using CEBRA [22] to characterize the underlying population dynamics. All these data establish the widespread spatial representation in the mouse cortex.

## Results

### The OGPI cranial window protocol: Workflow and quantitative benchmarks

**Fig. 1.**
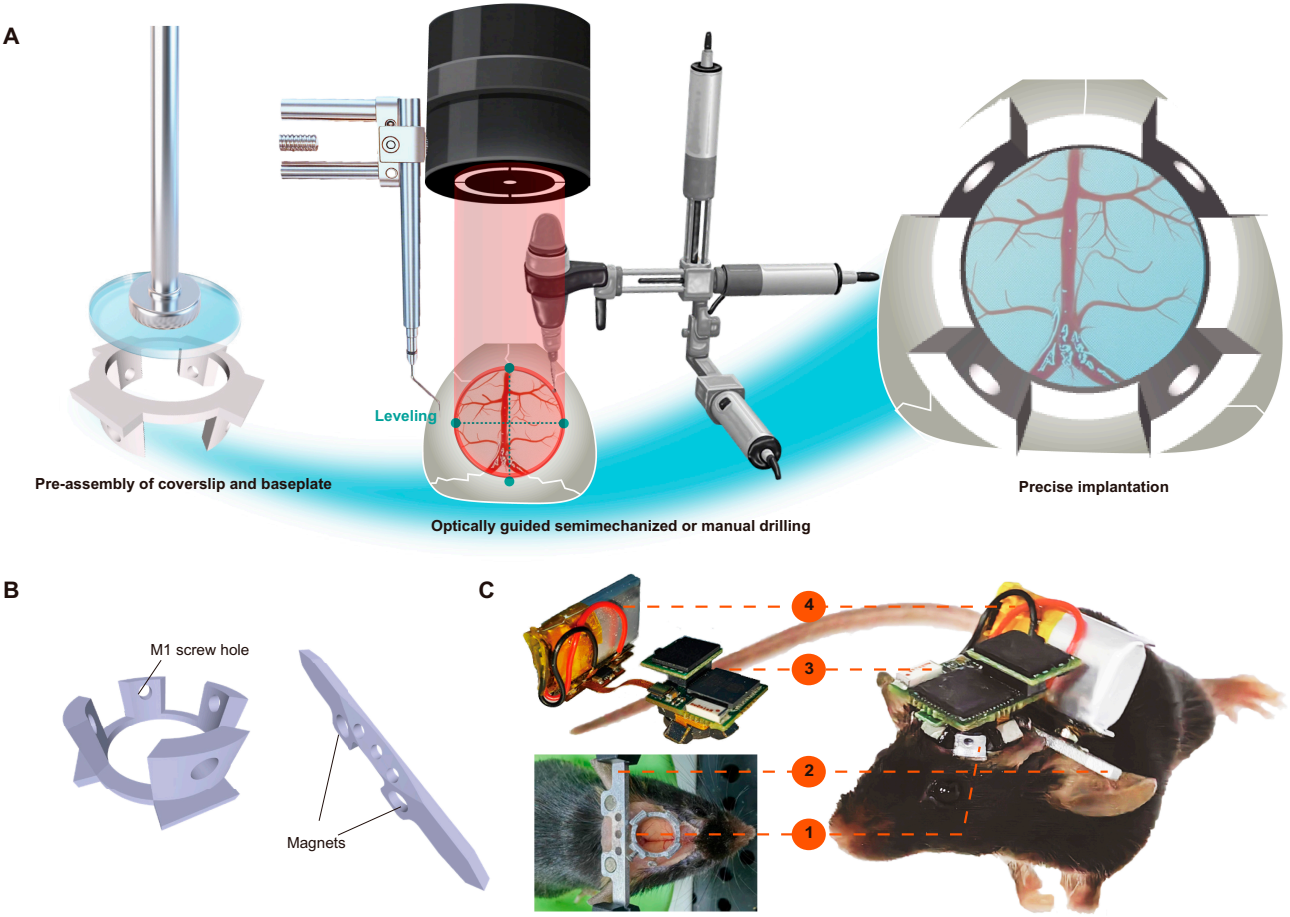
Overview of optically guided and pre-assembled implantation (OGPI) cranial window design and manufacturing. (A) Schematic diagram of OGPI cranial window establishment. (B) 3-dimensional (3D) designs for the cranial window of head-mounted microscopy. Top: a headplate with 4 M1 screw holes; the window-hole diameter was 7 mm. Bottom: a headbar with two 3-mm-diameter holes for embedding magnets. (C) Location 1 and location 2 show the headplate and the headbar implanted onto the mouse head; location 3 and location 4 show the head-mounted microscope and the battery.

**Fig. 2.**
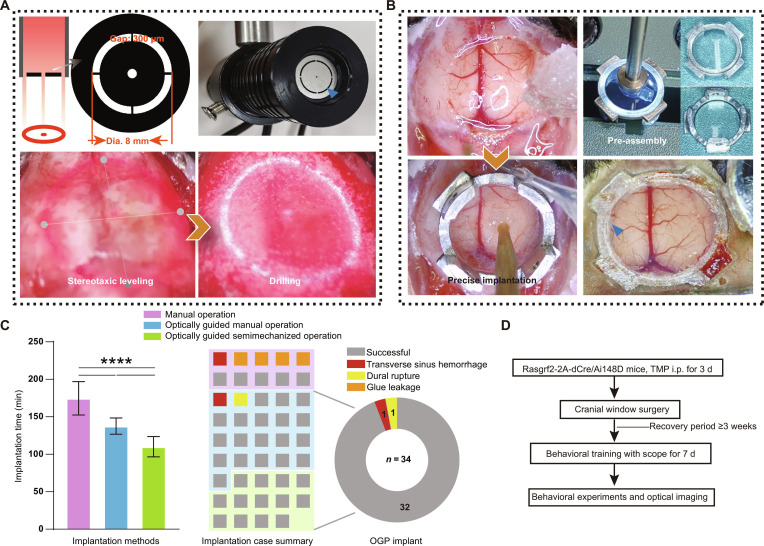
The OGPI optical guider design, cranial window conditions, and performance statistics. (A) Top: detailed design of the optical guider, a custom tool featuring a collimated light source with an annular filter at its tip. Diameter of the circular: 8 mm. Gap size: 0.3 mm. Bottom: the optical guider worked together with a stereotaxic instrument for leveling and cranial groove drilling. (B) Left: 8-mm-diameter circular craniotomy with preserved integrity of underlying meninges and dural sinuses. Precisely defined craniotomy dimensions allowed the glass component to fit within the circular opening and rest on the dural surface, while the metal baseplate was seated flush against the surrounding skull rim. Right top: the cover glass (8 mm diameter) was clamped through a custom-made magnetic gripper. Ultraviolet (UV) glue was applied on the metal baseplate bottom. Right bottom: the metal component was removed after clearing the dental cement above; the edge of the glass showed precise insertion without infection. (C) Left: durations of different implantation methods. Middle: a total of 44 operations are presented, namely, 10 manual implantations without optical guiding, 21 manual implantations with optical guiding, and 13 semimechanical implantations with optical guiding. Right: 34 OGPI cases showed no glue leakage with a 32/34 success rate. (D) Workflow of the mice processing pipeline. Data are presented as mean ± standard deviation (SD); *n* = 10, 21, and 13; one-way analysis of variance (ANOVA); ****P < 0.0001. i.p., intraperitoneal.

In this study, we developed an integrated pipeline for creating a large cranial window compatible with head-mounted miniaturized microscopes. This protocol utilizes optically guided semiautomated drilling to achieve precise implantation of the cranial window (Figs. 1 and 2A and B and Fig. S1). To overcome planarization challenges in large-field imaging, the coverslip is pre-assembled to the metal baseplate using ultraviolet (UV) adhesive, thereby ensuring the proper positioning of the head-mounted miniaturized microscope and achieving high-quality imaging across the entire field of view (FOV) (Fig. 2B and Fig. S2).

For accurate cranial opening, we designed a custom guiding tool incorporating a collimated light source with an annular filter at its tip to project an 8-mm ring onto the skull surface (Fig. 2A, top). Mounted on a stereotaxic frame, this tool projects a sharply defined 8-mm ring onto the mouse skull surface to direct drilling. Leveling with a Z-shaped probe relative to the projected ring enabled depth-fixed mechanical drilling by an RWD 71000 system [23] (Fig. 2A, bottom). Using the OGPI method, we showed that the edge of the glass was precisely inserted into the mouse skull without infection after the removal of the metal component 30 weeks post-implantation (Fig. 2B, right bottom).

A single skilled surgeon performed 44 implantations, namely, 10 manual conventional operations and 34 OGPI operations (21 manual operations with optical guiding and 13 semiautomated operations with optical guiding). The OGPI method significantly reduced surgery time compared to nonguided manual operations, with the shortest duration observed in the semiautomated group (*P* < 0.0001, one-way analysis of variance [ANOVA], *n* = 10, 21, and 13, Fig. 2C). More importantly, the OGPI method achieved a high success rate (32/34), and all OGPI cases showed that no unmatched window caused glue leakage, which could influence the imaging (Fig. 2C and Table S1).

For imaging, we employed Rasgrf2-2A-dCre/Ai148D mice, which allow the trimethoprim-induced expression of GCaMP6f in cortical layer 2/3 neurons (Fig. 2D). Both the baseplate and headbar fabricated from aluminum alloy and the actual photograph of a miniscope assembled onto the OGPI cranial window are shown (Fig. 1B). The microscope is mounted onto the baseplate, which features lateral screws for secure attachment. The headbar enables temporary head immobilization during the mounting procedure and incorporates 2 magnet holes posteriorly for battery attachment in wireless systems (Fig. 1C).

Detailed operation procedures are comprehensively demonstrated (Fig. S3 and Movie S1). Moreover, troubleshooting procedures for the OGPI method, which address issues such as optical window fractures in group-housed mice due to inter-male aggression, and headbar attraction due to magnetic polarity misalignment, are provided (Fig. S4).

### OGPI cranial windows support long-term, multimodal cortical imaging

**Fig. 3.**
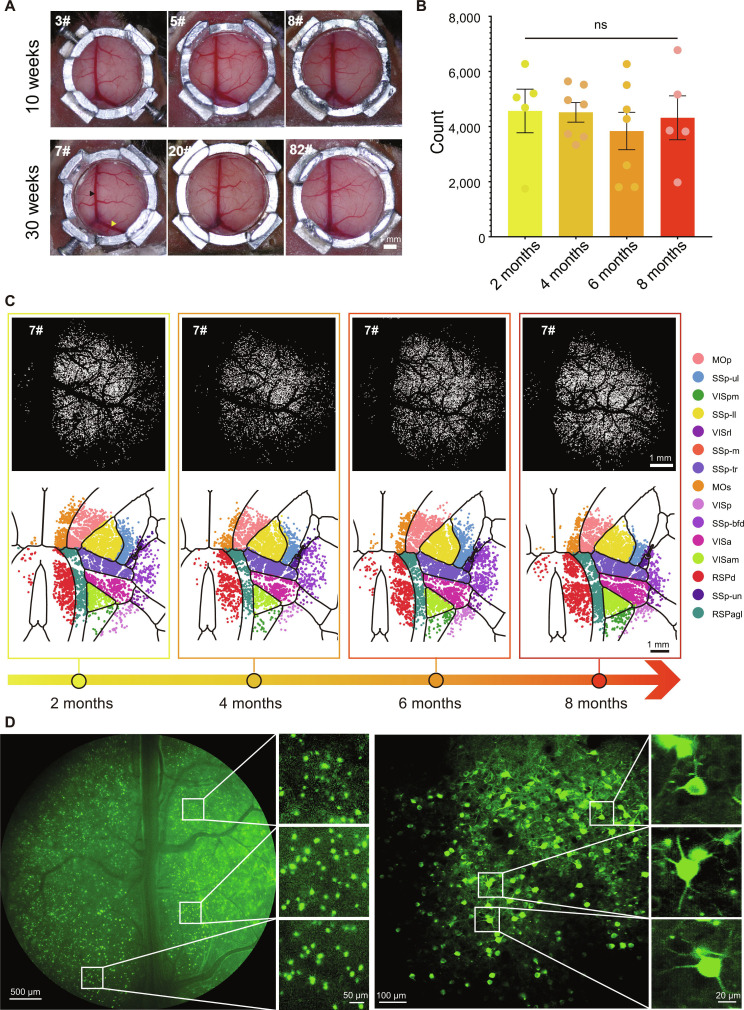
Quality assessment of large cranial windows over the long term through cortical calcium imaging by head-mounted, wide-field, and 2-photon microscopes in vivo. (A) Clarity of the large cranial windows across the superior sagittal and the transverse sinuses 30 weeks after implantation. Mouse IDs are shown in the top left corner of each panel. (B) Statistics of neural counts in the field of view (FOV) at 2, 4, 6, and 8 months after implantation. (C) Representative chronic neural calcium imaging in the same mouse over 8 months after cranial window implantation (7# mouse). (D) Left: cortex neural calcium imaging through wide-field microscopy. Right: subcellular structures including dendrites through 2-photon microscopy (20# mouse, 30 weeks after implantation). Data are presented as mean ± standard error of the mean (SEM); *n* = 5, 7, 7, and 5 mice; one-way ANOVA; *P* > 0.05. Abbreviations and their corresponding full names for cortical brain regions are shown in Materials and Methods. ns, not significant.

We introduced long-term monitoring of the OGPI cranial windows and demonstrated sustained structural integrity and optical transparency over 30 weeks post-implantation (Fig. 3A). To evaluate the consistency of cortical neural imaging over time, we conducted in vivo imaging in mice using a head-mounted miniaturized microscope at 2, 4, 6, and 8 months postsurgery. All animals were head-fixed under dark conditions in a familiar restraint apparatus while neuronal activity was recorded. Quantitative analysis demonstrated no significant differences in neural counts across post-implantation time points (*P* > 0.05, one-way ANOVA, *n* = 5 to 7 each group; Fig. 3B). Representative calcium imaging and cortical spatial maps from one mouse illustrated stable neuronal registration throughout the period (Fig. 3C). Additionally, we realized neuronal tracking across 3 different time points in one mouse after careful adjustment of the focal plane (day 1, day 3, and day 20). The result showed a substantial overlap in neuron positions, confirming that neurons can be tracked across different time points through the OGPI window, and the signal-to-background ratio of the OGPI windows across 8 months showed that there exists no significance between the 4 groups (Fig. S5). To anatomically align these calcium images with the Allen Brain Atlas [24], we also performed intrinsic signal imaging through the OGPI cranial window over the somatosensory cortex corresponding to the left hind paw and the tail. The dark regions observed in the intrinsic imaging (ISI) maps reliably indicated the functional areas representing the left hind paw and the tail (Fig. S6A to C). Moreover, the OGPI cranial window enabled high-resolution imaging across multiple modalities: benchtop wide-field microscopy captured large-scale cortical calcium imaging, while 2-photon microscopy resolved subcellular structures such as dendrites, even at 30 weeks post-implantation (Fig. 3D). Furthermore, we successfully performed CX3CR1^GFP^ imaging of both cortical and hippocampal regions, an achievement that relies critically on the pre-assembly and precise implantation capabilities of our system (Fig. S7). Moreover, we demonstrated the mechanical strength of the cranial window under 4 extreme behavioral scenarios that induce severe motion, namely, struggling, rotation, circling, and grand mal seizures (Fig. S8 and Movies S2 to S5).

### OGPI cranial windows preserve natural locomotor and spatial cognitive functions

Head-mounted miniaturized microscopes have enabled the recording of neural dynamics in freely moving animals [12,25]. However, the surgical implantation of large cranial windows that span multiple brain regions, including the motor cortex, somatosensory cortex, retrosplenial cortex, and midbrain, potentially affects motor behavior and limits experimental applicability. Therefore, we evaluated the impact of the OGPI cranial window on locomotor and spatial cognitive functions to validate its suitability for experiments that require free movement and spatial navigation.

**Fig. 4.**
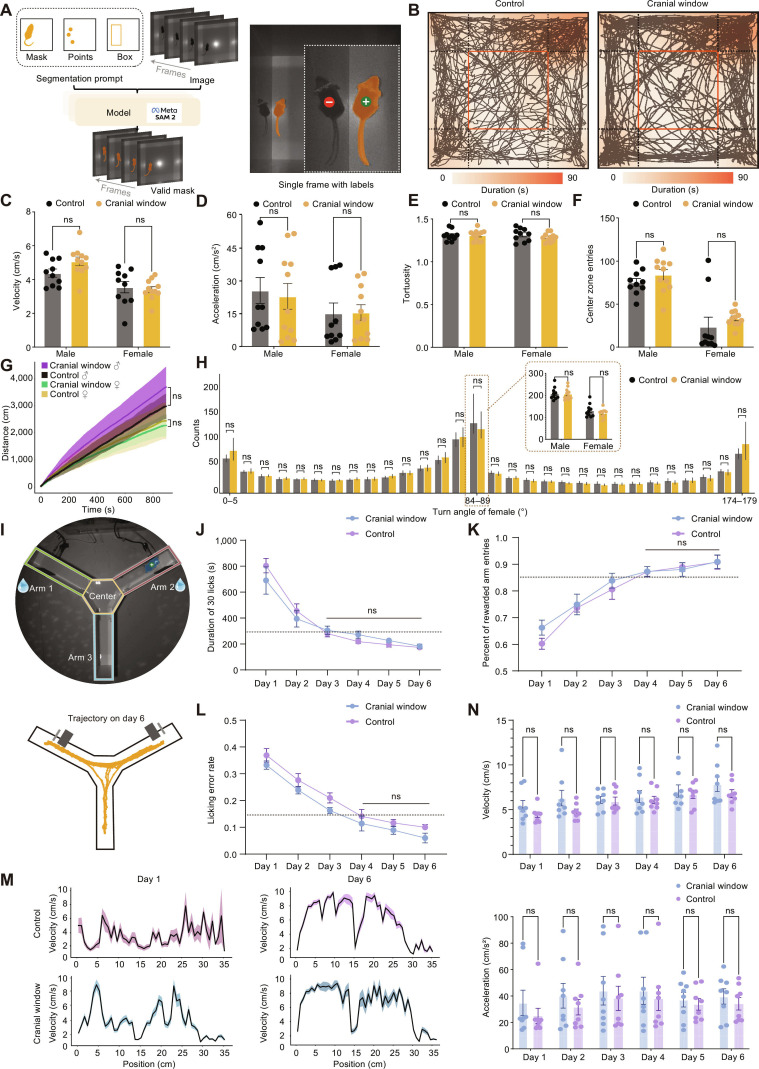
Behavioral assessments of mice with an OGPI cranial window. (A) Identification and segmentation of mice in an open-field arena using Segment Anything Model 2 (SAM 2)-based machine learning. (B) Sample trajectory heatmaps during 15 min for the cranial window group vs. the control group. (C) Distribution of the mean locomotion speed. (D) Distribution of the mean acceleration. (E) Distribution of the mean tortuosity. (F) Distribution of the mean center entries. (G) Accumulated distance in a 15-min open-field test (OFT). (H) Distribution of counts in different turn angle intervals. (I) Experimental setup of the Y-maze navigation paradigm. Top: identification and segmentation of mice in the Y-maze using SAM 2-based machine learning. Two waterspouts were located in arm 1 and arm 2 and pumped water alternatively. Bottom: a trajectory example on training day 6. (J) Learning curve: time to 30 effective licks’ cost. (K) Learning curve: the ratio of reward arm entries to empty arm entries during the 30-lick task period. (L) Learning curve: the error licking rate during the task. (M) Example velocity curves during navigation trials on training day 1 and training day 6. (N) Distribution of velocity and acceleration across the 6 training days. Data are presented as mean ± SEM; male: *n* = 8/group, trials = 30/d/mouse; *t* test; ns, *P* > 0.05.

We first compared 15-min open-field locomotion between mice with OGPI cranial windows and intact controls. Using Segment Anything Model 2 (SAM2)-based machine learning model, we objectively extracted postural kinematics and movement parameters in a 40 × 40 cm arena (Fig. 4A). No significant differences were observed between the cranial window and control groups in either sex across all measured parameters: accumulated distance traveled, velocity, acceleration, tortuosity, anxiety-related center entries, and turn angle distributions (*P* > 0.05, *t* test, male or female; cranial window: *n* = 11; control: *n* = 10; Fig. 4B to H).

To assess spatial cognitive function, we designed a self-paced Y-maze navigation paradigm under dark conditions using an alternating reward schedule. Two waterspouts were separately placed in the reward arms; approach toward an infrared sensor triggered water delivery (20 μl per activation). This design encouraged mice to actively and repeatedly shuttle between the 2 reward sites, facilitating efficient neural data acquisition across multiple uninterrupted trials without human intervention (Fig. 4I).

After water restriction (to 85% initial body weight), the cranial window group and control group underwent training. Learning curves were constructed based on multiple performance metrics: time to complete the first 30 valid licks, ratio of entries into reward arms versus those into the empty arm, and error lick rate. The results revealed that the learning curves between the cranial window and control groups were highly similar, with no significant differences (*P* > 0.05, *t* test, *n* = 8/group; Fig. 4J to L). From day 3 to day 6, task completion time stabilized below 300 s with no intergroup differences (*P* > 0.05, one-way ANOVA, *n* = 8/group; Fig. 4J). From day 4 to day 6, reward arm entries exceeded 85% and error lick rates fell below 15%, with no differences between groups (*P* > 0.05, one-way ANOVA, *n* = 8/group; Fig. 4K and L). Collectively, these results indicate that at least 3 training days were required to achieve stable performance.

Trained mice exhibited precise approaches toward reward spouts. We found that the navigation velocity between reward sites shifted from a disordered pattern to a stereotyped trajectory-aligned profile (Fig. 4M), indicating enhanced spatial memory of reward locations. Further, we analyzed general locomotor parameters including speed and acceleration across training days and found no differences between the cranial window and control groups, ruling out the influence of motor capabilities on navigation efficiency (*P* > 0.05, *t* test, *n* = 8/group; Fig. 4N).

### Decoding spatial navigation from widespread cortical activity

**Fig. 5.**
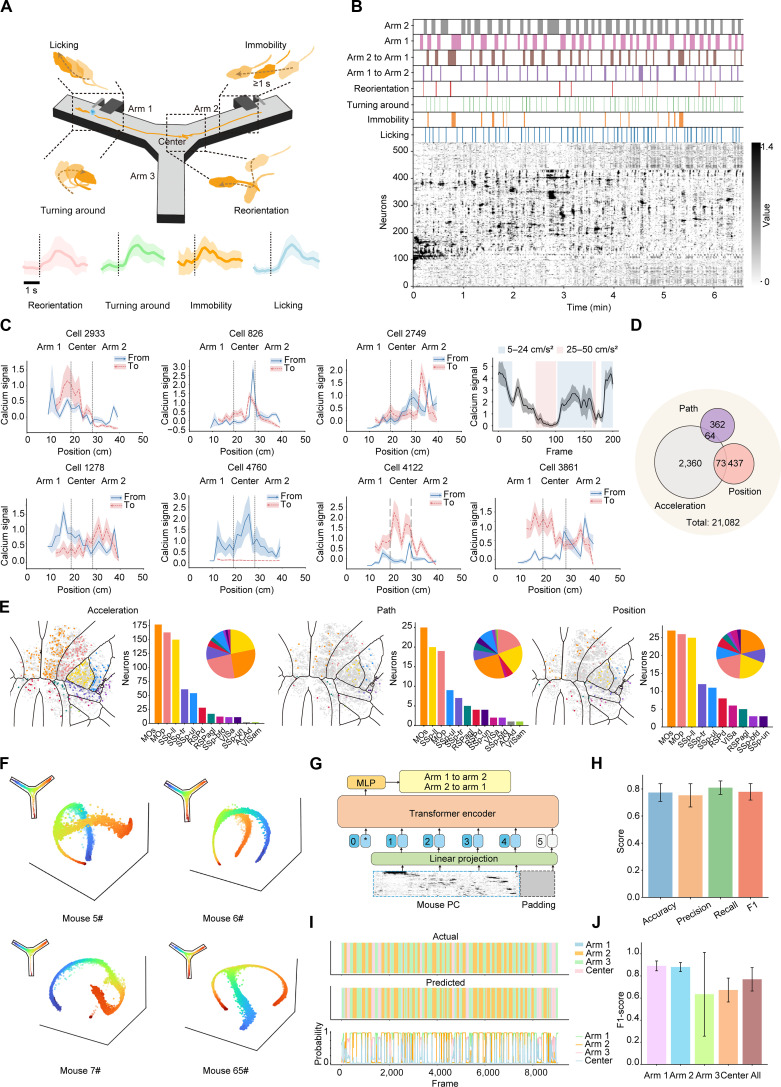
Cortical neural dynamics during the navigation task in the Y-maze. (A) Simultaneous behavioral and neural activities during navigation in the Y-maze. Bottom: Examples of cells tuning reorientation, turning around, immobility, and licking. (B) Behavioral labels within navigation trials and the raster plot of dorsal cortical neurons during navigation. (C) Examples of position-tuned (top left, cell 2933, cell 826, and cell 2749), acceleration-tuned (top right, average of 10 neurons), and path-tuned cells (bottom, cell 1278, cell 4760, cell 4122, and cell 3861). (D) Distribution of the 3 types of tuned cells (21,082 cells from 4 mice were recorded). (E) Cross-cortical distribution of the 3 types of neurons. (F) CEBRA-behavior position manifolds (*n* = 4 mice). (G) Decoding model architecture with a transformer encoder. (H) Decoding performance for transitions between 2 regions of the Y-maze, including accuracy, precision, recall, and F1-score (*n* = 233 trials from 4 mice). (I) Representative results of position decoding from one mouse. (J) Classifier performance metrics (F1-scores) for predicting the positions (*n* = 4 mice). Data are presented as mean ± SD; *n* = 233 trials from 4 mice. MLP, multilayer perceptron. PC, principal component.

We further conducted simultaneous recording of cortical neural activity and navigation behavior in trained mice, using a head-mounted miniaturized microscope combined with an OGPI cranial window. Diverse behavioral labels and their associated typically tuned neurons were identified—including reorientation, turning around, immobility, and licking events (Fig. 5A and Movie S6). Visualization of behavioral events alongside raster plots of cortical activity revealed potential organization of neural activity during Y-maze navigation (Fig. 5B).

We focused our analysis on navigation periods, defined as traversals between the 2 reward areas: from the initiation of movement in arm 1 to arrival at the reward zone in arm 2, before water delivery was triggered (Movie S6). We observed 3 types of neurons, neurons that responded to specific positions (510/21,082 cells), neurons that responded to specific accelerations (2,497/21,082 cells), and neurons that responded to path-locked positions (426/21,082 cells), across 233 trials from 4 mice (Fig. 5C and D). Conjunctive tuning was observed in acceleration-tuned neurons, with some exhibiting additional tuning to position and others to path-locked position (Fig. 5D). Acceleration-tuned neurons, path-tuned neurons, and position-tuned neurons (510/21,082 cells) all showed “salt-and-pepper” distribution across the cortex (Fig. 5E). We utilized 2 decoding networks to probe the functional significance of these neural populations in behavioral decoding (Fig. S9). These analyses revealed that acceleration-tuned neurons made a relatively larger contribution to the models’ overall decoding performance. To rigorously dissociate position and acceleration variables, we employed multiple decomposition analyses (Fig. S10). These analyses confirmed that acceleration-tuned and position-tuned neurons are distinct populations that contribute differentially to neural encoding.

We further analyzed the representation of spatial information in the cerebral cortex from a population-level neuronal perspective. Applying CEBRA dimensionality reduction to neuronal ensembles revealed neural manifolds that accurately reflected the physical space (Fig. 5F). We then used population neural activity to decode both the animals’ paths and their instantaneous locations. First, we predicted the animals’ transitions between the reward arms of the Y-maze (arm 1 → arm 2 or arm 2 → arm 1), which yielded favorable results (77.33% ± 6.55% accuracy, 75.29% ± 8.60% precision, 80.91% ± 4.98% recall, and 77.86% ± 6.09% F1-score, mean ± SD, *n* = 4 mice), indicating that cortical neuronal populations encode paths information during navigation (Fig. 5G and H). We then predicted the animal’s position across 4 regions: arm 1, arm 2, arm 3, and the center; the predicted outcomes closely matched the actual locations (84.98% ± 4.09% accuracy, 85.47% ± 4.08% precision, 84.98% ± 4.09% recall, and 84.46% ± 3.90% F1-score, mean ± SD, *n* = 4 mice), indicating that the position is encoded in the population activity of cortical neurons (Fig. 5I and J). To confirm that our results were not artifacts of stereotyped, nonlocomotory behaviors, we performed a control analysis by rerunning the decoder after meticulously identifying and removing all neural data frames corresponding to water-licking periods at the reward ports. The performance of the decoder showed almost no difference before and after removing the related frames, which strengthens the conclusion that the population activity contains a robust representation of spatial location that is not solely dependent on motor signals associated with specific reward-related behaviors (Fig. S11).

## Discussion

In this study, we introduce the OGPI method, a novel protocol that enables long-term large-field imaging using head-mounted miniaturized microscopes. We provide open-source designs and detailed surgical tutorials to facilitate broad adoption. The OGPI method ensures precise craniotomy, shortens operation time, and achieves a high success rate (32/34) for stable implantations spanning dural sinuses. The window maintains optical clarity for over 8 months, enabling chronic neural monitoring. Using this method, we successfully acquired and analyzed cortical neuronal activity during behavior, demonstrating its robustness for large-field calcium imaging throughout extended experiments.

Several technical challenges were addressed in developing the OGPI method. First, achieving single-cell resolution across a large FOV requires strict focal alignment. Unlike benchtop microscopes, head-mounted miniaturized microscopes are rigidly fixed onto the skull via baseplates, eliminating the possibility of focal adjustment by tilting the sample. Although existing 2-photon head-mounted systems such as Mini2P offer a substantial imaging depth (~240-μm z-scanning) within a moderate FOV, they typically involve baseplate attachment and adjustment after window implantation, guided by imaging feedback [11]. We found that manually attaching the baseplate after creating a large cranial window makes it difficult to establish a uniform focal plane across the entire FOV. To overcome this, we pre-assemble the coverslip with the metal baseplate using a custom magnetic holder before the implantation. This ensures that the optical axis of the microscope remains approximately perpendicular to the imaging surface, thereby enabling large-FOV imaging at single-cell resolution.

The second challenge was achieving precise implantation of the pre-assembled unit. Precise placement is essential for sealing (preventing glue leakage and infection) and for providing sufficient mechanical stability to support the microscope during unrestrained behavior. Although a limited number of studies have introduced automated robotic systems for craniotomy [16,23,26], the majority of current protocols still depend on fiducial markers and surgeon expertise [17,18,27–29]. Robotic approaches remain technologically complex and cost prohibitive, whereas conventional manual methods are often unstable and time-consuming. Here, we utilized parallel light for guidance through a custom optical guider mounted on the standard stereotaxic frame. This guide is compatible with both automated drilling by RWD 71000 [23] and manual drilling. Furthermore, the optical guider can be readily adapted for any required window shape and size through different aperture masks, highlighting the broad applicability of the approach.

The third challenge involved the safe removal of the bone flap. Large cranial windows carry an increased risk of dural rupture and sinus hemorrhage if the bone flap is lifted directly [14,30]. In this study, we recommend that during the separation of the bone flap from the dura mater, a gelatin sponge should be applied to initiate dissection between the dural sinuses and the lambdoid suture. The underlying dura is tightly adherent to the cranial sutures [31,32], creating high tension. Releasing this area first effectively reduces tension and helps preserve the integrity of the dura and sinuses. In summary, the core principle of OGPI lies in integrating parallel light guidance, pre-assembly, and a secure head-restraint system, which together enable its broad applicability. This integrated approach is essential for achieving stable, high-quality imaging, particularly when using large-FOV miniature microscopes.

A potential concern with cranial windows is their possible impact on neural function. For example, abnormal auditory brain stem response suggests that the optical cranial window slightly influences the auditory function [33]. In this study, the OGPI cranial window covered the motor cortex and retrosplenial cortex and crossed the transverse sinus, potentially extending to midbrain regions, all involved in motor function [34,35]. This led us to question whether mice with OGPI windows can have normal activity and be competent for behavioral tasks. Therefore, we conducted comprehensive behavioral assays and confirmed that mice with OGPI implants exhibit normal locomotor and spatial cognitive abilities.

Having established the long-term stability of the OGPI cranial window, we next leveraged this method to investigate cortical function during Y-maze navigation. To mitigate the challenges of neuronal drifting and recording instability, which require the acquisition of sufficient trials and continuous, undisturbed monitoring during behavioral sessions [36], we implemented an alternative water reward system. A similar strategy can make animals actively and repeatedly do the navigation task [21]. We collected 233 qualified navigation trials of simultaneous neural and behavioral data from 4 task-proficient Rasgrf2-2A-dCre/Ai148D transgenic mice. We identified cortical neurons that were tuned to position or path widely distributed throughout the cortex, in a “salt-and-pepper” pattern. Although spatial tuning during animal’s locomotion mostly focused on the hippocampal–entorhinal system [21,37–39], growing evidence suggests its broader cortical distribution. For example, head direction cells and angular head velocity cells exhibit “salt-and-pepper” distributions in the retrosplenial cortex [40,41]. Spatially tuned neurons such as place, grid, and head direction cells are identified in the somatosensory cortex [42]. Place and head direction cells are also distributed across the visual cortex regions [25,43]. These earlier studies indicate the widespread distribution of spatial information in the cortex. Our findings, which provide a large-scale and systematic survey, are consistent with and markedly extend this emerging view by demonstrating the widespread nature of these representations. These earlier studies each involved several distinct cortical regions related to spatial information in the cortex. In contrast, the OGPI method enabled us to simultaneously map the distribution of spatially tuned neurons across several cortical areas. Our findings, which offer a large-scale and systematic investigation, support the distributed representation of spatial information across the cerebral cortex and further extend this understanding by demonstrating the widespread nature of such representations during the navigation task.

We also analyzed acceleration signals, which integrate multisensory cues from vestibular, proprioceptive, and visual systems [44]. The vestibular system is known to play a critical role in supporting spatial navigation circuits involving the hippocampal–entorhinal network [45,46]. Our results demonstrate that acceleration-tuned neurons are widely distributed across cortical regions during motion in darkness, consistent with broad cortical representations of vestibular signals as mapped by evoked-response studies [47]. Notably, we identified subpopulations of cells exhibiting conjunctive tuning to both linear acceleration and spatial information. This aligns with the prevailing paradigm wherein continual self-motion updates generate a dynamically updated sense of self-location [44]. Our results suggest that conjunctive representations of acceleration with position or path exist in the cortex. However, the specific neural computations underlying these integrative representations, as well as the relative contributions of cortical and subcortical structures, remain to be elucidated.

Finally, while tuning specificity at the single-neuron level is informative, it does not necessarily reflect the encoding strategy at the population level. We further used a decoder or a classifier utilizing cortical neural population activity to predict the location (arm 1, arm 2, arm 3, or center) and which paths the animal was traversing (arm 1 → arm 2 vs. arm 2 → arm 1). The high decoding accuracy indicates that information about location and path is encoded in the population activity of cortical neurons.

In conclusion, our results reveal the widespread cortical representation of spatial navigation and self-motion signals. These findings were enabled by the stable, long-term neural access provided by the OGPI method, which represents a robust and accessible platform for future investigations of cortical function in behaving animals. This study has several limitations that should be acknowledged. First, despite adherence to the principle of separating the bone flap along sutures, manual removal of the bone flap still poses a potential risk of dural or sinus damage. Second, the OGPI window poses challenges for the coverage of lateral cortical surfaces, such as the insular cortex, primarily due to constraints imposed by the inherent size of the mouse skull. Third, a larger sample size would be necessary to enable more in-depth neural analysis during navigational tasks. In particular, neural interaction between the cortex and the hippocampus warrants further investigation in future studies.

## Materials and Methods

### Animals

All animal experiments were conducted in compliance with animal welfare guidelines and were approved by the Tsinghua University Institutional Animal Care and Use Committee. The animals were provided by the Tsinghua University Laboratory Animal Center. Rasgrf2-2A-dCre/Ai148D transgenic mice were generated by crossing Rasgrf2-2A-dCre mice (JAX Stock #022864) with Ai148 (TIT2L-GC6f-ICL-tTA2)-D transgenic mice (JAX Stock #030328). In this strain of mice, the Cre expression is predominantly restricted to layers 2/3 of the cortex. CX3CR1^GFP^ mice (JAX Stock #005582) were employed for simultaneous imaging of microglial cells. Male mice aged 8 weeks and female mice aged 10 weeks were used. Animals were housed under controlled conditions (temperature: 22 to 24 °C; humidity: 55%) with a 12-h light/dark cycle and provided ad libitum access to food and water except during specific experimental protocols. Transgenic mice received trimethoprim lactate (15 mg/ml, 0.01 ml/g, intraperitoneal) induction per day for 3 consecutive days, 3 weeks before the imaging. The unilateral labyrinthectomy mouse model was built referring to a previous study [48]. The seizure mouse model was built through pentylenetetrazol (PTZ, intraperitoneal, 35 mg/kg, Sigma-Aldrich) kindling as previously described [49].

### Pre-assembly of the glass–metal baseplate

The baseplate and headbar were custom-designed to accommodate the wireless head-mounted microscope. Further details can be found in Fig. 1. One day prior to surgery, the baseplate was fabricated as follows: first, the metal baseplate was secured to the workbench. A custom-made magnetic gripper was then used to grip the glass coverslip (8.0 mm × 0.17 mm). The stereotaxic apparatus was carefully adjusted to ensure precise alignment between the glass coverslip and the top of the metal baseplate. Finally, the assembly was fixed using UV-curable adhesive (Loctite, AA 3311) and cured under UV light for 5 min. The custom headbars were prepared by permanently affixing 2 micro-circular magnets (3 mm × 2 mm) into the machined receptacles with UV-curable adhesive. Both sides should be coated with glue, and the side facing up should be as thin and light as possible to facilitate the later fixation of the microscope battery. This assembly was then exposed to UV light for 5 min to cure the adhesive.

### Cranial window surgery

All surgical instruments were sterilized before the operation. Anesthesia was induced and maintained with isoflurane (1% to 1.5%, RWD, R500) while the mouse was positioned on a heating pad (37 °C). Eye ointment was applied to both eyes, which were then covered with aluminum foil. Following skin preparation and disinfection, the cranial skin was removed to expose the skull. The superficial fascia was cleared away, and portions of the temporal and occipital muscles were dissected and excised. The optical guider, a custom collimated light source, worked together with the stereotaxic instrument for marking the cranial window dimensions and leveling the circular. The skull was drilled using RWD 71000 or manually drilled along the circular groove until fissures became visible. The bone flap was elevated as described (Movie S1 and Fig. S3); gelatin sponge was used to separate the flap from the underlying dura. For hippocampus exposure [50], the cortex was removed guided by the optical guider (Fig. S7). The pre-assembled glass–metal unit was placed onto the cortex with its glass surface on the dura and its metal component on the skull. The stereotaxic instrument needle was lowered until complete apposition between the glass and cortical surface was achieved. Initial fixation was performed using biological adhesive (3M Vetbond), followed by reinforcement with cyanoacrylate glue (Loctite 435). A 1-mm-diameter hole was drilled on the left side of the window, and a skull screw was implanted therein. The surrounding muscle and tissue were sealed with biological adhesive, followed by attachment of the headbar to the upper occipital region using cyanoacrylate glue. All exposed skull surfaces and skin margins were then completely covered with dental resin cement. Ceftriaxone sodium trihydrate (0.25 mg/g, Aladdin) was injected intraperitoneally to prevent infection. For analgesia, meloxicam (1 mg/kg, Tsinghua University Laboratory Animal Center) was administered subcutaneously for 3 d postsurgery. Mice recovered on heating pads until they regained mobility, followed by single housing during the postoperative recovery period. All mice after cranial window implantation received 3-week recovery before the experiment.

### Behavioral experiments

All animals underwent head-mounted microscope adaption training and handling habituation for 1 week to reduce experimenter-induced stress.

#### Open-field test

The open-field arena measured 40 cm × 40 cm referring to a previous study [51]. Prior to the start of the experiments, mice were acclimated to the testing room for 1 h. The arena was thoroughly cleaned with 75% ethanol before each trial to eliminate odor and other potential confounding factors. For testing, mice were placed at a designated starting position facing the center of the arena and allowed to explore freely for 16 min while their behaviors, movement trajectories, and cortical neuronal calcium signals were simultaneously recorded. An infrared high-speed camera (FLIR) was used to record the video at 10 frames per second (fps) above the arena. After excluding the initial 30-s adaption period, the behavior was analyzed for 15 min. Locomotor performance was quantitatively assessed through detailed analysis of accumulated distance traveled, velocity, acceleration, tortuosity [25], anxiety-related center entries, and head rotation distributions across angular bins [40].

#### Y-maze navigation task

All mice were individually housed and water-restricted until a stable body weight was maintained at 85% of baseline. The Y-maze apparatus consisted of three 30-cm arms, with one designated as the start arm. The distal ends of the other 2 arms were equipped with a pump system (Tushui, TS006GMB) consisting of 2 liquid delivery ports programmed to dispense 20-μl aliquots of water upon alternate triggering of the infrared sensors. Experiments were conducted under dark conditions with supplementary infrared illumination. An infrared high-speed camera (FLIR) was used to record the video at 19 fps above the maze. The maze was thoroughly cleaned with 75% ethanol before each trial to eliminate odors and other confounding factors, and mice were acclimated to the testing room for 1 h simultaneously. At trial initiation, each mouse was positioned facing the Y-maze center within the start arm, after which the partition was removed to permit free exploration. We analyzed the first 30 successful lick events across a 6-d training period. Behavioral and neuronal activity during exploration and reward consumption were recorded, with navigation efficiency (the time used to complete the first 30 valid licks, the ratio of entries into the reward arms versus those into the empty arm, and the proportion of error licks relative to total licks during this period) serving as the primary metric for spatial cognition and navigational ability assessment. Licking was defined as licking events with water delivery, and dry lick was defined as licking events without water delivery [52]. Immobility was defined as the movement speed of a mouse below 2 cm/s for ≥1 s [53]. Turnaround was defined as mouse turns around 180°. Reorientation was defined as the mouse redirecting its heading from one maze arm to another while within the central zone [54]. All behaviors were manually annotated and marked by 2 blinded observers using self-made software (Fig. S12). All basic locomotor metrics including speed and acceleration were acquired through SAM 2-based machine learning [55].

#### Tail suspension test

The mouse underwent the tail suspension test as previously described [56]. The mouse was suspended by its tail 60 cm above the floor. A 10-mm-diameter tube was affixed to the mouse’s tail to prevent it from climbing its own tail. The mouse’s behavior was recorded by a video camera for 6 min, and neural imaging was recorded simultaneously.

#### Head rotation test

The mouse head was rotated referring to a previous study [40]. Briefly, the mouse head was fixed on a servomotor-driven rotation stage (Hengyu, D80C). The mouse was rotated back and forth for 120° at 80°/s 5 times. The calcium imaging was simultaneously recorded.

### Calcium imaging

For calcium imaging, Rasgrf2-2A-dCre/Ai148D mice were used, which allow trimethoprim-induced expression of GCaMP6f in cortical layer 2/3 neurons. All imaging rooms were kept dark and quiet with no external disturbances. All tested mice were acclimated to the room for 1 h before imaging. For wide-field imaging, we employed a ×2 magnification system equipped with an MVPLAPO 2XC 0.5 NA objective, using 488-nm laser excitation at 10 fps for 2 min. For 2-photon microscopy, the metal cover over the cranial window was carefully removed before imaging. A drop of deionized water was applied as immersion medium for the water-immersion objective (Olympus XLPlan N 25×/1.05 NA). Excitation was performed at 920 nm, with image acquisition at 15 fps and a resolution of 1,024 × 1,024 pixels, and data were acquired for 2 min. All data were saved in TIFF format and subsequently processed using ImageJ. For head-mounted microscopy, a custom-built wireless miniaturized microscope, an upgraded version of our previous large-field head-mounted microscope [12,57], was mounted on the OGPI cranial window for calcium imaging at 10 Hz, with the focus adjusted using 3-dimensionally printed spacers (Movie S1). Multiple mice were imaged at different post-implantation time points (2, 4, 6, and 8 months). For simultaneous imaging during behavior tests, mice underwent 1-h daily acclimation to the microscope for 1 week prior to the test to minimize device-induced interference.

### Intrinsic imaging

In this study, ISI techniques were employed for brain region segmentation in mice after the postoperative recovery period referring to a previous study [41]. All experiments were conducted in a quiet and dark environment. Detection was performed using a tactile swab stimulator modified based on the Thorlabs GVS211/M galvanometer (with a vibration frequency of 10 Hz), coupled with a red-light-emitting diode head-mounted microscope (wavelength: 630 nm). During the experiments, mice were head-fixed, maintained under slight anesthesia with 0.5% to 1% isoflurane, and placed on a heating pad. The focal plane of the microscope was adjusted to 100 μm below the clearest field of the blood vessel’s view. First, the left hind paw of the mice was exposed, and microscope recording and timing were started simultaneously. After 10 s of baseline recording, a pre-activated vibrating rod was applied to stimulate the left hind paw for 15 s, followed by a 20-s rest period. This cycle was repeated 3 to 5 times to complete data collection for the left hind paw. Subsequently, the identical experimental protocol was followed, with the only modification being that the stimulation site was changed to the tail for data collection. The intrinsic image was the difference between the projection of stimulation images and baseline images. Next, the obtained ISI and blood vessels were aligned with the Allen Brain Atlas [24], and then these were co-registered to the calcium images to complete neuronal image registration (Fig. S6).

The abbreviations and their corresponding full names for cortical brain regions are as follows: SSp-bfd, primary somatosensory area, barrel field; SSp-un, primary somatosensory area, unassigned; SSp-tr, primary somatosensory area, trunk; SSp-ll, primary somatosensory area, lower limb; SSp-ul, primary somatosensory area, upper limb; SSp-m, primary somatosensory area, mouth region; VISrl, rostrolateral visual area; VISam, anteromedial visual area; VISpm, posteromedial visual area; VISp, primary visual area; RSPv, retrosplenial area, ventral part; RSPd, retrosplenial area, dorsal part; RSPagl, retrosplenial area, lateral agranular part; VISa, anterior area; MOp, primary motor area; and MOs, secondary motor area.

### Behavior and neuronal activity analyses

#### SAM 2-based behavior analysis

We used SAM 2 [55] to perform semantic segmentation on mice, and obtained the center of the mask as the centroid of the mice. The checkpoints used were sam2.1_hiera_base_plus. The segmentation prompt included 3 types: mask, points, and box. Moreover, we distinguished the positive and negative samples of the mice.

#### Neuronal segmentation pipeline

Captured raw data from the miniaturized mesoscope were first motion-corrected using the open-source NoRMCorre algorithm in nonrigid mode [58]. The motion-corrected movies were processed by the recently developed DeepWonder package [59] to extract reliable neuronal segments and signals. DeepWonder was fine-tuned to the detailed characteristics of the miniaturized mesoscope modality following the guidelines in the DeepWonder article [59].

#### Tortuosity

Momentary tortuosity was quantified as the ratio between the cumulative path length (*L*(*t*)) and the straight-line distance (*D*(*t*)) connecting the window boundaries, using a symmetric sliding window of ±1.25 s around each recording time point. To reduce noise, the *x* and *y* coordinates of the mask centroid, *X*(*t*) and *Y*(*t*), respectively, were smoothed with a 0.5-s moving average. The path length *L*(*t*) within each window was obtained by integrating the frame-to-frame displacement *d*(*t*).

$$L\left(t\right)\;=\;\sum_{t-1.25\;s}^{t+1.25\;s}d\left(t\right)$$ (1)

The straight-line distance *D*(*t*) was defined as the Euclidean distance from the start frame (*t* − 1.25 s) to the end frame (*t* + 1.25 s). The momentary tortuosity at time point *t* was then calculated as *T*(*t*) = *L*(*t*)/*D*(*t*). Analyses were restricted to epochs of active locomotion, with running defined by a speed threshold corresponding to the mean of the 75th percentile speeds across all control recordings (11 males and 10 females). For these periods, the 75th percentile values of tortuosity were extracted from frames exceeding the speed threshold.

#### Turn angle

The Cartesian coordinates of the centroid of the mouse were determined for each frame, and the turning angle was quantified by calculating the change in trajectory in the horizontal plane using coordinates from 3 consecutive frames [40]. The distribution of turn angles over a set distance was then compared between control and cranial window groups.

#### Significance test of 3 neuron types

##### Acceleration-tuned

We first binned instantaneous acceleration into 2 categories: “low acceleration” (5 to 24 cm/s^2^) and “high acceleration” (25 to 50 cm/s^2^). To avoid potential confounds from nonlocomotory behaviors (e.g., drinking and slight movements), we deliberately excluded time points corresponding to negative accelerations (deceleration) or very low accelerations (0 to 5 cm/s^2^). Neurons were then identified as acceleration-tuned if they exhibited a statistically significant response (*P* < 0.05) to these 2 acceleration categories.

##### Position-tuned

We spatially divided the Y-maze into 3 distinct regions: “arm 1”, “center”, and “arm 2”. We then identified trials where the mouse was in each region. Neurons were classified as position-tuned if their activity showed a significant modulation (*P* < 0.05) corresponding to 1 or more of these 3 static spatial zones.

##### Path-tuned

To identify neurons selective for the animal’s trajectory, we segmented each round-trip trial into 3 distinct phases based on progression: “starting” (the origin arm), “central” (the intermediate area), and “terminal” (the destination arm). Neurons were classified as path-tuned if their activity was significantly modulated (*P* < 0.05) by one or more of these specific phases of the journey.

#### CEBRA [22]

To evaluate the performance in the Y-maze experiment, we trained the CEBRA model, which is based on the neural signals recorded in the cortex. We used the official CEBRA implementation without any modifications and followed the standard analysis pipeline provided in the authors’ demonstration notebook, including default model settings and training procedures (https://cebra.ai/docs/demo_notebooks/Demo_hippocampus.html). The model architecture has an offset of 10 and an output embedding dimension of 3 and is trained using cosine distance, with 5,000 training iterations and a batch size of 512.

#### Neural decoding

To evaluate whether cortical population activity could be used to predict the outcome of Y-maze test encounters, we implemented a transformer-based decoding model. The neural activity from each trial was organized into matrices (neurons × time) and reduced to a fixed spatial dimension using principal component analysis, retaining the top 256 components to account for the variability in the number of recorded neurons. The resulting representations were treated as temporal sequences, with each time point encoded as a 256-dimensional vector. Sequences were zero-padded to a uniform length of 196 tokens, where padding vectors were handled as special tokens similar to those used in natural language processing.

For model input, the [CLS] token was used to represent the transition path of the mouse—either from arm 1 to arm 2 or from arm 2 to arm 1. Each 256-dimensional vector was linearly projected to a 128-dimensional embedding, followed by the addition of position embeddings. The [CLS] embedding was then used by a multilayer perceptron classifier to predict the trial outcome (arm 1 → arm 2 or arm 2 → arm 1).

The dataset consisted of recordings from 4 mice (total trials *n* = 233), divided into training and validation sets with an 8:2 ratio. Model optimization was performed using the AdamW optimizer (initial learning rate 1 × 10^−4^, weight decay 1 × 10^−4^), with training for 30 epochs. A cosine annealing schedule was applied to adjust the learning rate, and dropout (0.3) was used in both attention and multilayer perceptron modules to mitigate overfitting. The model was trained with cross-entropy loss to optimize classification accuracy.

#### LSTM decoding model

At the same time, we also used a deep learning model based on bidirectional long short-term memory (LSTM) [60] to decode the position information of the mice.

Calcium traces were segmented into nonoverlapping 2-s windows, each containing *N* recorded neurons over time bins. To avoid temporal data leakage, the step size between consecutive windows was set to twice the window length, ensuring that adjacent samples did not overlap. The position label at the end of each window served as the target for decoding. Data were split into 70% for training and 30% for validation.

For LSTM, the decoder first projected each neural population vector into lower-dimensional embedding via a linear layer and layer normalization. Temporal features were extracted using one-dimensional convolutions to capture short-time-scale patterns, followed by bidirectional LSTM to model long-range dependencies. The deep network was optimized using cross-entropy loss and the Adam optimizer (learning rate = 0.001, weight decay = 1 × 10^−5^) with a learning rate scheduler that reduced the learning rate by a factor of 0.5 when the validation loss plateaued. Training was performed for 50 epochs with a batch size of 16. Model performance was evaluated using accuracy, recall, precision, and F1-score.

We also implemented 2 LSTM networks to decode behavioral variables from neural activity. The first model was a regression network trained to predict the mouse’s continuous future trajectory (*X* and *Y* coordinates). The model input consisted of neural activity from the preceding 1.5 s (15 time points), and the target output was the animal’s movement trajectory 0.5 s (5 time points) into the future. The second model was a classifier network designed to decode the animal’s current, discrete location. The Y-maze was spatially divided into 5 distinct zones. The model used neural activity from a 10-frame window to classify the mouse’s corresponding location within that same window.

For both models, the dataset was randomly partitioned into training (80%) and testing (20%) sets. Models were trained using a batch size of 64 and a learning rate of 5 × 10^−4^ for 80 epochs.

#### General linear model

To statistically quantify the distinct contributions of position-tuned neurons and acceleration-tuned neurons to animals’ movement, we implemented an encoding-focused general linear model. We modeled the movement trajectory of the animal on the *x*-axis at time *t*, trajectory *x*(*t*), as a linear function of the animal’s concurrent position-tuned neurons and acceleration-tuned neurons.

The model is described by the following equation:

$$\text{Trajectory}\;x\left(t\right)\;=\;\beta_{0}\;+\;\beta_{1}\;\times \;\text{Position}\left(t\right)\;+\;\beta_{2}\;\times \;\text{Acceleration}\left(t\right)\;+\;\epsilon$$ (2)

#### *d*′ value

To most rigorously dissociate the 2 variables, we conducted a *d*′ analysis, adapted from sensory coding literature [61]. This analysis directly tests whether a neuron can discriminate between acceleration states while the spatial location is held constant.

We first isolated all trials that passed through the same spatial region (e.g., regions 1 and 2.). Then, we split these “location-matched” trials into 2 groups based on the animal’s instantaneous acceleration: a “high-acceleration” group (25 to 50 cm/s^2^) and a “low-acceleration” group (5 to 24 cm/s^2^). Finally, we calculated the *d*′ value for acceleration-tuned neurons, which measures the separability of the neural response distributions between these 2 groups.

The *d*′ value was computed using the standard formula:

$$\frac{\mu_{\text{high}}\;-\;\mu_{\mathrm{low}}}{\sqrt{0.5\;\times \;\left(\sigma_{\text{high}}^{2}\;+\;\sigma_{low}^{2}\right)}}$$ (3)

where $\mu_{\text{high}}$ and $\sigma_{\text{high}}^{2}$ are the mean and variance of the neural response during high-acceleration trials, respectively, and $\mu_{\mathrm{low}}$ and $\sigma_{\mathrm{low}}^{2}$ are the mean and variance during low-acceleration trials, respectively, all within the same spatial bin.

### Statistical analysis

All data in this study were analyzed using GraphPad Prism (version 10.0) and SPSS (version 27.0). Prior to statistical analysis, data were subjected to tests for normality and homogeneity of variance. If both assumptions were satisfied, the *t* test was applied; if either assumption was violated, nonparametric tests were used instead. All figures were generated using GraphPad Prism (version 10.0) or Python, with the final layout/formatting by Adobe Illustrator.

## Data Availability

The computer-aided design model for the OGPI cranial window and the custom code and software are available on GitHub at https://github.com/lemon123457/OGPI. Any additional information required to reanalyze the data reported in this paper is available from the lead contact upon reasonable request.

## Supplementary Materials

Supplementary 1 Figs. S1 to S12 Tables S1 and S2 Movies S1 to S6 References [27,62–78]
