# Supplementary material for: The Optically Guided and Pre-assembled Implantation Cranial Window Reveals Cortical Spatial Representations during Navigation
Source: Research (Wash D C). 2026 Jan 15;9:1072. doi: 10.34133/research.1072 (PMC12804600; doi:10.34133/research.1072)
Supplement: Supplementary 1 — Figs. S1 to S12 Tables S1 and S2 Movies S1 to S6 References [27,62–78] [file research.1072.f1.zip › S2_Table.docx]

| **This is a statistical summary of the OGPI method alongside other techniques. Different methods are often engineered for specific applications.** | | | | | | | | | | | | |
| --- | --- | --- | --- | --- | --- | --- | --- | --- | --- | --- | --- | --- |
| **-** | **Applicable Microscope Types** | **Trans-sinus Capability** | **Compreh-ensive operation series** | **Surgical success rate（%）** | **Duration** | **Cranial window positioning methods** | **Window pictures** | **Craniotomy area** | **Optical window** | **Articles** | **Authors** | **Others** |
| 1 | Wireless Head-mounted | Transverse sinus: Yes;  Superior sagittal sinus: Yes | Yes | 94.12 | 8 months | Parallel light guidance | 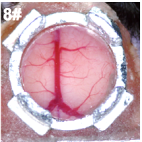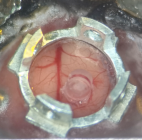 | Large round area，＞45 mm^2^ (Dia. 8 mm) | Glass with metal | This job | This job | pre-assembly and precise，multiregion |
| 2 | Head-mounted | Transverse sinus: No;  Superior sagittal sinus: Yes | Not mentioned | Not mentioned | Not mentioned | Traditional stereotaxic positioning | 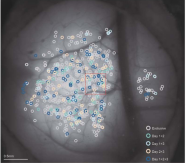 | Large round area，13 mm^2^ (Dia. 4 mm) | Glass | Yuanlong Zhang et al. Nature Biomedical engineering.2024^[12]^ | Dai et al. | - |
| 3 | Tabletop | Transverse sinus: No;  Superior sagittal sinus: Yes | Not mentioned | Not mentioned | Not mentioned | Traditional stereotaxic positioning | 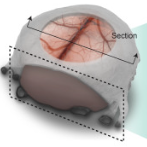 | Large trapezoid area，＞45 mm^2^ | Glass | Yuanlong Zhang et al. Cell.2024^[3]^ |  | 3D |
| 4 | Tabletop | Transverse sinus: No;  Superior sagittal sinus: Yes | Not mentioned | Not mentioned | Not mentioned | Traditional stereotaxic positioning | 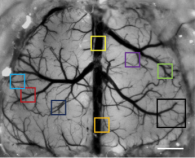 | Large trapezoid area，＞45 mm^2^ | Curved Glass | Hao Xie et al. Nature Biomedical engineering. 2024^[62]^ |  | - |
|  |  |  |  |  |  |  | Scale bar=1 mm |  |  |  |  |  |
| 5 | Tabletop | Transverse sinus: No;  Superior sagittal sinus: Yes | Not mentioned | Not mentioned | Not mentioned | Not mentioned | 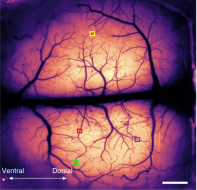 | Large trapezoid area，＞45 mm^2^ | Glass | Jingtao Fan et al. Nature photonics.2019^[63]^ |  |  |
|  |  |  |  |  |  |  | Scale bar=1 mm |  |  |  |  |  |
| 6 | Tabletop | Transverse sinus: No;  Superior sagittal sinus: Yes | Yes | 93.5 | 337 days | a robot that uses surface profiling to guide a computer numerical controlled (CNC) mill | 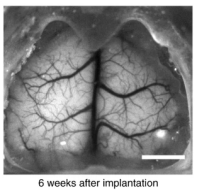 | Large trapezoid area，＞45 mm^2^ | Curved Transparent polymer | Leila Ghanbari et al. Nature Communications , 2019^[64]^ | Suhasa B. Kodandaramaiah et al. | curved polymer |
|  |  |  |  |  |  |  | Scale bar=2 mm |  |  |  |  |  |
| 7 | Head-mounted | Transverse sinus: No;  Superior sagittal sinus: Yes | Not mentioned | Not mentioned | Not mentioned | a standard rodent stereotax (Model 900LS; Kopf) or an automated robotic surgery platform, the Craniobot | 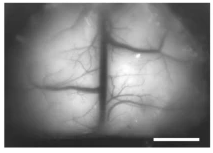 | Large trapezoid area，＞45 mm^2^ | Curved Transparent polymer | Mathew L. Rynes et al. Nature Methods, 2021^[65]^ |  | robotic surgery platform |
|  |  |  |  |  |  |  | Scale bar=2 mm |  |  |  |  |  |
| 8 | Tabletop | Transverse sinus: No;  Superior sagittal sinus: Yes | Yes | Not mentioned | Not mentioned | Craniobot | 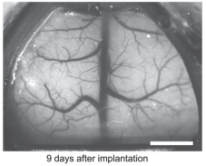 | Large trapezoid area，＞45 mm^2^ | Glass | Mathew L Rynes et al. Nature Protocol, 2020^[26]^ |  | Craniobot |
|  |  |  |  |  |  |  | Scale bar=2 mm |  |  |  |  |  |
| 9 | Tabletop | Transverse sinus: No;  Superior sagittal sinus: No | Not mentioned | near 100% success rates | Not mentioned | CV-Craniobot with OCT device for position acquisition | 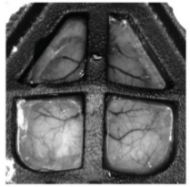 | Large trapezoid area，＞45 mm^2^ | Glass (make a robot to do the craniotomy) | Zahra S. Navabi et al. Science Advances, 2025^[16]^ |  | CV-Craniobot with OCT device |
| 10 | Tabletop | Transverse sinus: Yes;  Superior sagittal sinus: Yes | Yes | Not mentioned | >6 months | Not mentioned | 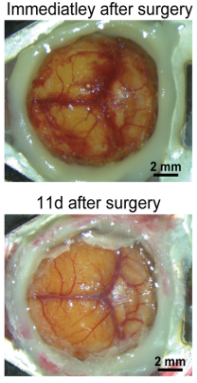 | Large round area，＞45 mm^2^ (Dia. 8 mm) | Fluoropolymer nanosheet and light-curable resin | Taiga Takahashi et al. Communications Biology, 2024^[14]^ | Tomomi Nemoto et al. | Fluoropolymer,cross sinuses |
| 11 | Tabletop | Transverse sinus: Yes;  Superior sagittal sinus: No | Not mentioned | upwards of 80-90% | 175 days | Traditional stereotaxic positioning and manual marking with marker pen | 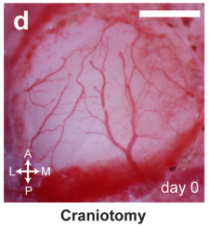 | Large round area, 19.6 mm^2^ (Dia. 5 mm) | Glass | Glenn J Goldey et al. Nature Protocol, 2014^[13]^ | Mark L Andermann | replaceable cranial window |
| 12 | Head-mounted | Transverse sinus: No;  Superior sagittal sinus: No | Yes | Not mentioned | Not mentioned | Traditional stereotaxic positioning | 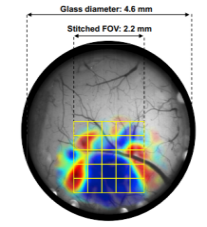 | Large round area, 16.6 mm^2^ (Dia. 4.6 mm) | Glass | Weijian Zong et al. Cell, 2022^[25]^ | May-Britt Moser, Edvard I. Moser | two-photon |
| 13 | Tabletop | Transverse sinus: Yes;  Superior sagittal sinus: No | Not mentioned | over 90% | 60 days | Traditional stereotaxic positioning + manual marking with marker pen | 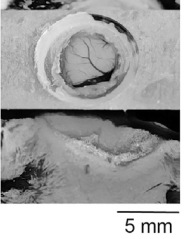 | Large round area, 19.6 mm^2^ (Dia. 5 mm) | Glass | Sigita Augustinaite et al. STAR Protocols, 2020^[18]^ | Sigita Augustinaite | - |
| 14 | Tabletop | Transverse sinus: No;  Superior sagittal sinus: No | Not mentioned | Not mentioned | Not mentioned | Traditional stereotaxic positioning | 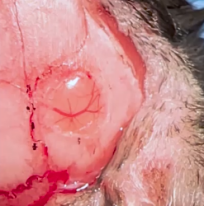 | Large round area,7.1 mm^2^ (Dia. 3 mm) | Glass | Ragunathan Padmashri et al. J Vis Exp.2021^[66]^ | Anna Dunaevsky | - |
|  |  |  |  |  |  |  | JOVE video |  |  |  |  |  |
| 15 | Tabletop | Transverse sinus: No;  Superior sagittal sinus: No | Not mentioned | Not mentioned | Not mentioned | Not mentioned | 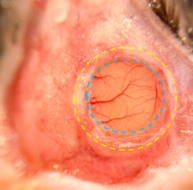 | Large round area, 15.9 mm^2^ (Dia. 4.5 mm) | Glass | Ikumi Oomoto et al. STAR Protocols, 2021^[28]^ | Masanori Murayama et al. | - |
| 16 | Tabletop | Transverse sinus: No;  Superior sagittal sinus: Yes | Not mentioned | Not mentioned | Not mentioned | Not mentioned | 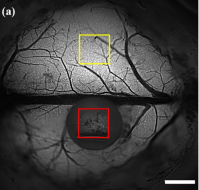 | Large round area, 38mm^2^ (Dia. 7mm) | Glass with microprism | Rujin Zhang et al. Biosensors, 2022^[67]^ | Jiangbei Cao et al. | microprism |
|  |  |  |  |  |  |  | Scale bar=1 mm |  |  |  |  |  |
| 17 | Tabletop | Transverse sinus: No;  Superior sagittal sinus: No | Not mentioned | Not mentioned | Not mentioned | Not mentioned | 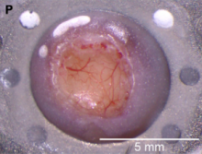 | Large round area, 7 mm^2^ (Dia. 3 mm) | Glass | Masayuki Sakamoto et al. STAR Protocols, 2022^[16]^ | Haruhiko Bito et al. | - |
| 18 | Tabletop | Transverse sinus: No;  Superior sagittal sinus: Yes | Not mentioned | 82% | 10 weeks | Traditional stereotaxic positioning | 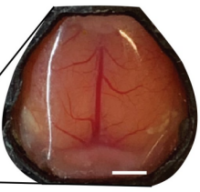 | Large trapezoid area，＞45 mm^2^ | Polymer(PMP) | Bradley J. Edelman et al. PLOS BIOLOGY, 2024^[68]^ | Emilie Mace et al. | compatible with fus |
| 19 | Tabletop | Transverse sinus: No;  Superior sagittal sinus: Yes | Not mentioned | Not mentioned | 9 months | Traditional stereotaxic positioning | 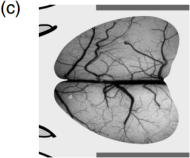 | Large trapezoid area，＞45 mm^2^ | Polymethylpentene(PMP) | Marine Tournissac et al. Neurophotonics, 2022^[69]^ | Serge Charpak et al. | compatible with fus |
| 20 | Tabletop | Transverse sinus: No;  Superior sagittal sinus: No | Not mentioned | Not mentioned | 10 weeks or longer | Traditional stereotaxic positioning and manual marking with marker pen | 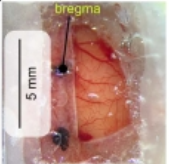 | Large rectangle area, 18 mm^2^ | PVDC | Satoshi Manita et al. J Vis Exp.2022^[17]^ | Kazuo Kitamura | - |
| 21 | Tabletop | Transverse sinus: No;  Superior sagittal sinus: Yes | Not mentioned | Not mentioned | 8 weeks | Not mentioned | 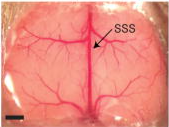 | Large trapezoid area，＞45 mm^2^ | Curved Glass | Tony Hyun Kim et al. Cell Reports. 2016^[6]^ | Mark J Schnitzer | Curved crystal Skull |
|  |  |  |  |  |  |  | Scale bar=1 mm |  |  |  |  |  |
| 22 | Tabletop | Transverse sinus: No;  Superior sagittal sinus: No | Not mentioned | Not mentioned | 15 weeks | Traditional stereotaxic positioning | 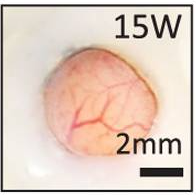 | Large round area, 19.6 mm^2^ (Dia. 5 mm) | PDMS | Chaejeong Heo et al. Sci Rep. 2016^[15]^ | Minah Suh | Repetitive penetration |
| 23 | Tabletop | Transverse sinus: No;  Superior sagittal sinus: Yes | Not mentioned | Not mentioned | 6 months | Traditional stereotaxic positioning and manual marking with marker pen | 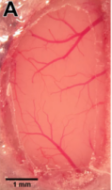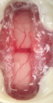 | Large rectangle area, 14 mm^2^ | PMP | Kıvılcım Kılıc et al. Frontiers in physiology, 2020^[70]^ | Kıvılcım Kılıc | - |
| 24 | Tabletop | Transverse sinus: No;  Superior sagittal sinus: No | Not mentioned | 72.73% | 22 weeks | Traditional stereotaxic positioning | 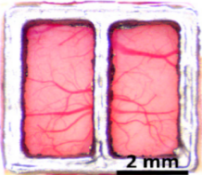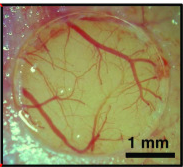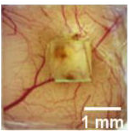 | Large rectangle area, Approx.20 mm^2^ | Titanium-PDMS | Nana Yang et al. Biosensors, 2022^[71]^ | Shengyong Xu et al. | - |
| 25 | Tabletop | Transverse sinus: No;  Superior sagittal sinus: No | Not mentioned | Not mentioned | 3 months | Traditional stereotaxic positioning | 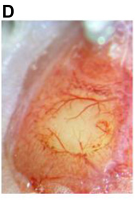 | Large round area, 7 mm^2^ (Dia. 3 mm) | Glass | Signe H. Mikkelsen et al. Frontiers in Neuroscience, 2022^[72]^ | Eugenio Gutiérrez-Jiménez et al. | - |
| 26 | Tabletop | Transverse sinus: No;  Superior sagittal sinus: No | Not mentioned | 71.42% | 16 weeks | Traditional stereotaxic positioning | 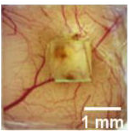 | square area, 9 mm^2^ | Glass with microprism | Qianru Yang et al. Biomaterials. 2021^[73]^ | Xinyan Tracy Cui | glass with prism |
| 27 | Tabletop | Transverse sinus: No;  Superior sagittal sinus: No | Not mentioned | Not mentioned | Not mentioned | Traditional stereotaxic positioning | 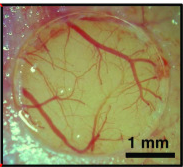 | Large round area, 7 mm^2^ (Dia. 3 mm) | Cover glass with electrode | Yin, R. et al. Neurophotonics. 2022^[74]^ | Lan Luan | cover glass with electrode |
| 28 | Tabletop | Transverse sinus: No;  Superior sagittal sinus: No | Not mentioned | Not mentioned | 6 weeks | Traditional stereotaxic positioning | 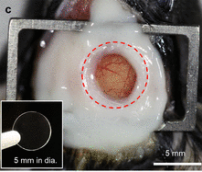 | Large round area, 19.6 mm^2^ (Dia. 5 mm) | PVA/PDMS | Jong Uk Kim et al.  ACS Appl Mater Interfaces. 2022^[75]^ | Tae-Il Kim et al. | - |
| 29 | Tabletop | Transverse sinus: No;  Superior sagittal sinus: No | Not mentioned | Not mentioned | several months | Traditional stereotaxic positioning | 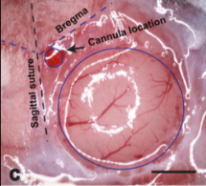 | Large round area, 13 mm^2^ (Dia. 4 mm) | Glass | Viviana Zuluaga-Ramirez et al. Fluids Barriers CNS, 2015^[76]^ | Yuri Persidsky | repeated intracerebral injections and simultaneous in vivo imaging of the mouse brain |
| 30 | Head-mounted | Transverse sinus: No;  Superior sagittal sinus: No | Yes | Not mentioned | Not mentioned | Not mentioned | 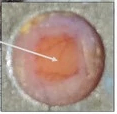 | A small square cranial window (6.25 mm2) | Glass | Zong, Weijian et al. Nature methods,2017^[77]^ | Heping Cheng et al. | - |
| 31 | Head-mounted | Transverse sinus: No;  Superior sagittal sinus: Yes | Not mentioned | Not mentioned | Not mentioned | 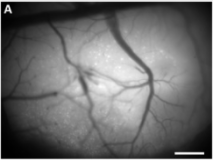Traditional stereotaxic positioning |  | A square cranial window (16 mm2) | Glass | Guo, Changliang et al. Science advances ,2023^[78]^ | Daniel Aharoni et al. | - |
|  |  |  |  |  |  |  | Scale bar=0.5 mm |  |  |  |  |  |
