# Supplementary material for: The Optically Guided and Pre-assembled Implantation Cranial Window Reveals Cortical Spatial Representations during Navigation
Source: Research (Wash D C). 2026 Jan 15;9:1072. doi: 10.34133/research.1072 (PMC12804600; doi:10.34133/research.1072)
Supplement: Supplementary 1 — Figs. S1 to S12 Tables S1 and S2 Movies S1 to S6 References [27,62–78] [file research.1072.f1.zip › sm.docx]

SUPPLEMENTARY MATERIALS


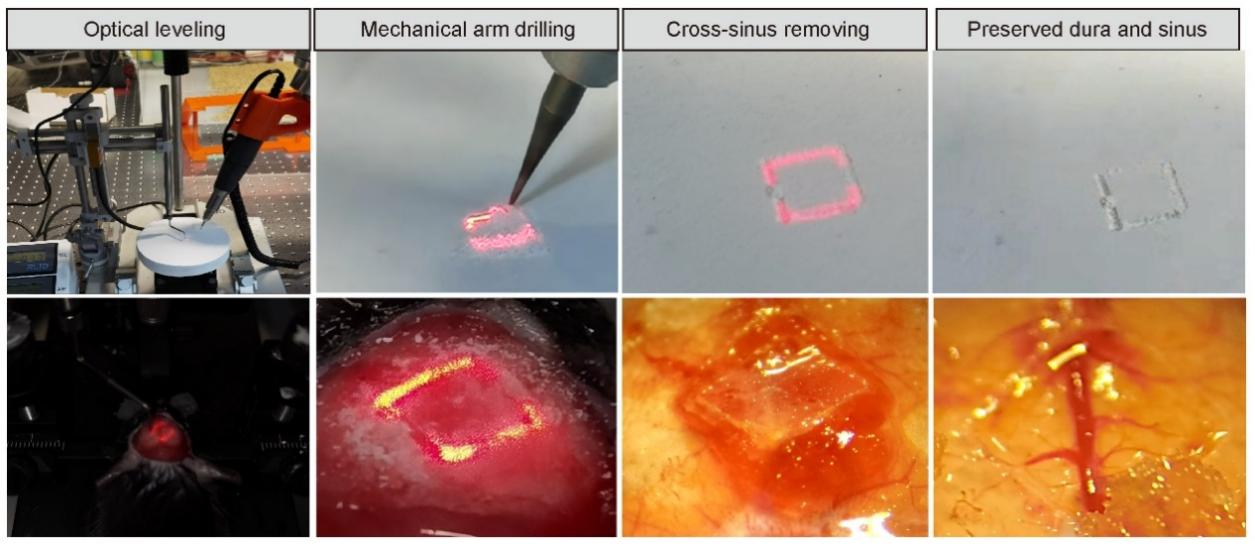


**Fig. S1. The OGPI method was integrated with automated workflows.** From left to right: leveling alongside the projected square shape (3×3 mm^2^); mechanical arm drilling; cross-sinus removing alongside the skull suture; the sura and sinus were well preserved.


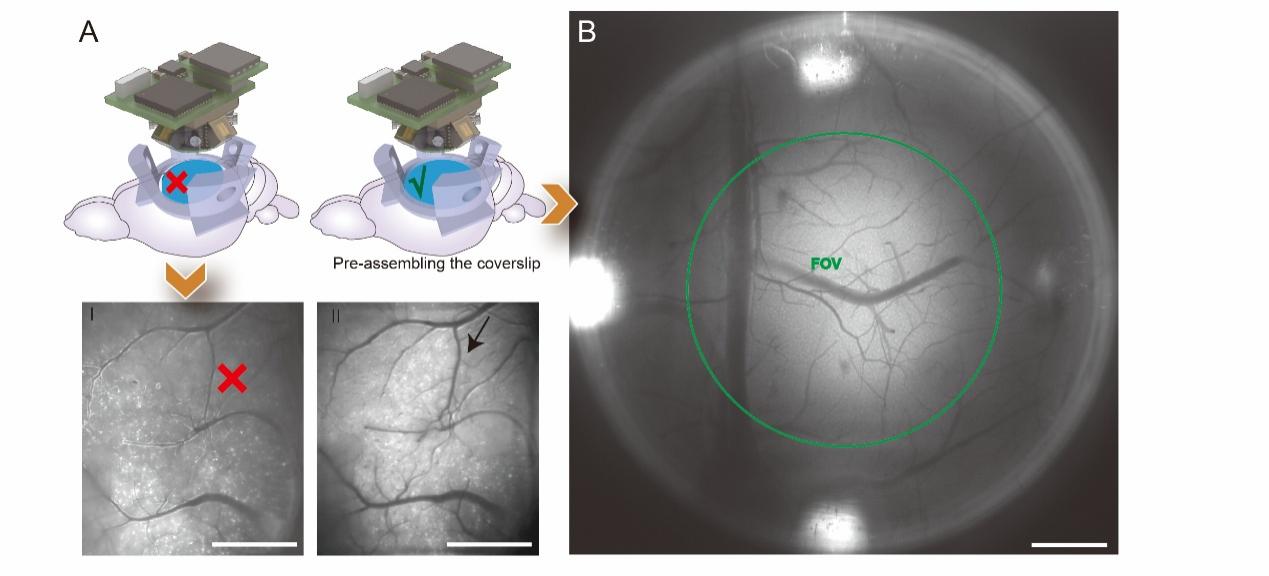


**Fig. S2. Pre-assembling of the glass-metal baseplate.** (A) Separately implanting the glass and the metal baseplate of the microscope showed affected imaging in FOV: top area of the image showed imaging blur after fixing the microscope (I) despite actual neural Ca²⁺ presence (II). (B) Pre-assembling of the glass and the metal components ensured the planarization across the FOV. Scale bars: 1 mm.


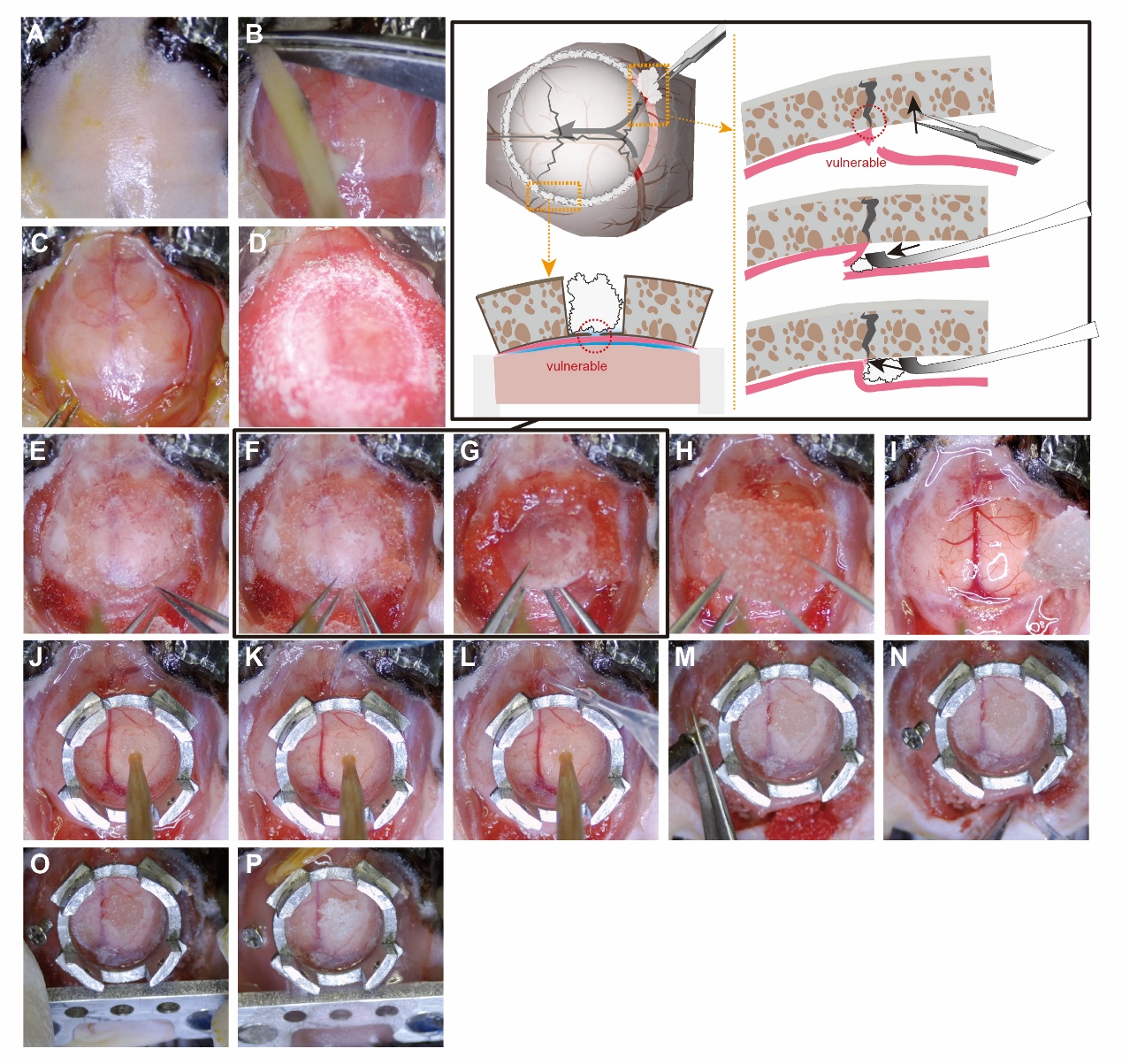


**Fig. S3. Step-by-step surgical procedures.** (A) Surgical field sterilization by iodophor. (B, C) The overlying tissue was removed to expose the cranial surface. (D) An 8mm-diameter circular groove was drilled guided by a specialized optical indicator. (E) Clearance of the debris in the right-down cranial opening (breakthrough zone). (F, G) Two essential skills for safe removal of large cranial across major dural sinuses: dural protection by inserting moist hemostatic sponge into the groove before shifting the bone flap; initiating dura detachment sequentially from the lambdoid suture to the sagittal suture along the suture lines above the blood sinuses. (H, I) The hemostasis and cleaning of the dura mater surface using moist sponge. (J) The coverglass with headplate was pressed appropriately onto the brain. (K) 3M glue was applied. (L) Loctite 435 glue was applied. (M) Drilling a nail into the skull for reinforcement. (N) The skin edge was attached to the skull surface by 3M glue. (O) A headbar was applied onto the skull. (P) Application of the dental cement. The edge of the metal part should be covered.

**
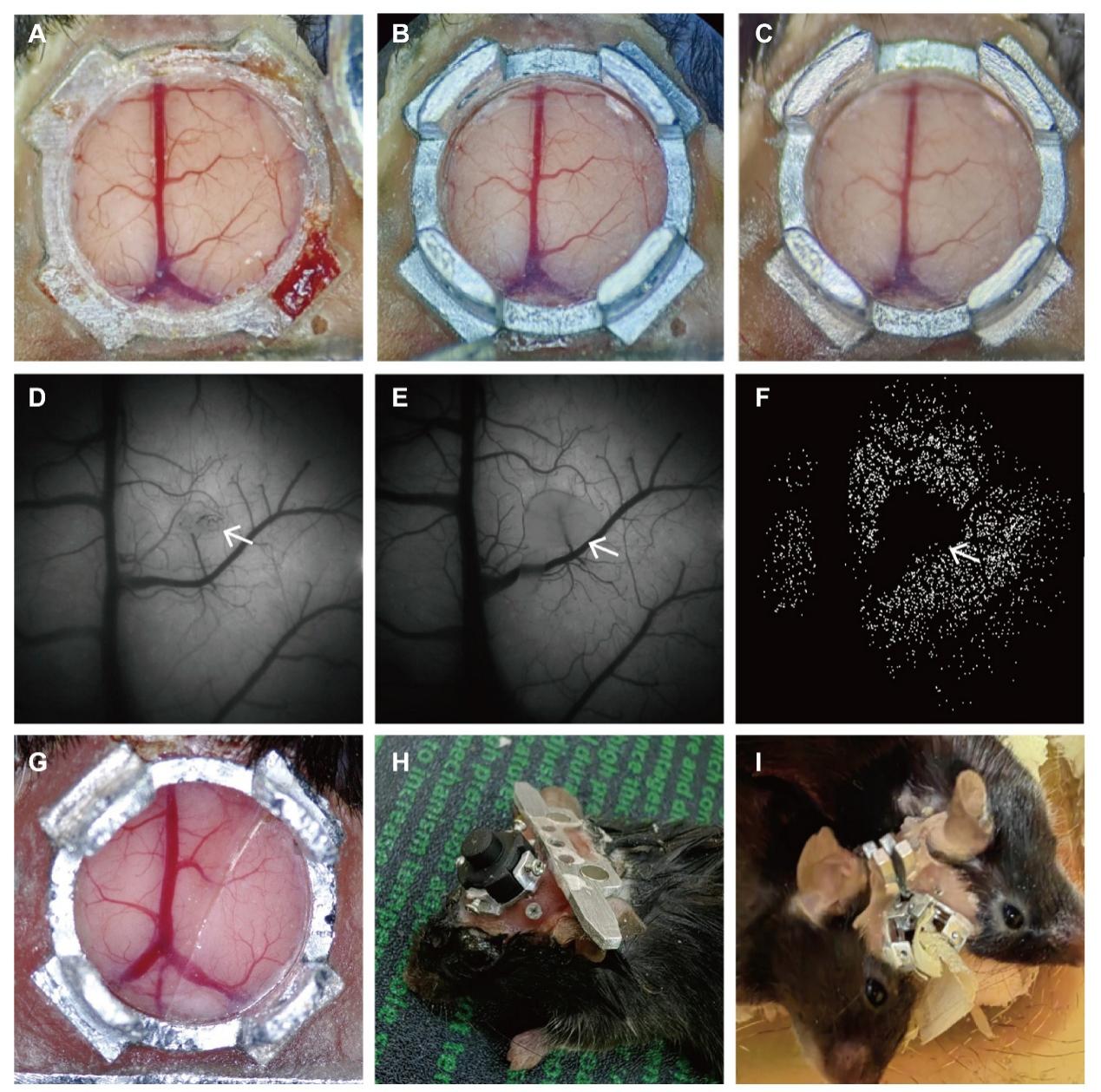
**

**Fig. S4. Trouble shooting.** (A-C) Replacement of the metal part. (D-F) Pressing the head-mounted microscopy too hard can lead to secondary bleeding, which trigger membranous hyperplasia around the affected area, ultimately resulting in the loss of calcium imaging in the region. (G) The large glass window from a male mouse housed in groups cracked. (H) Covering the window with latex to protect the glass. (I) Opposite magnets lead to head attraction between two mice in daily breeding management.


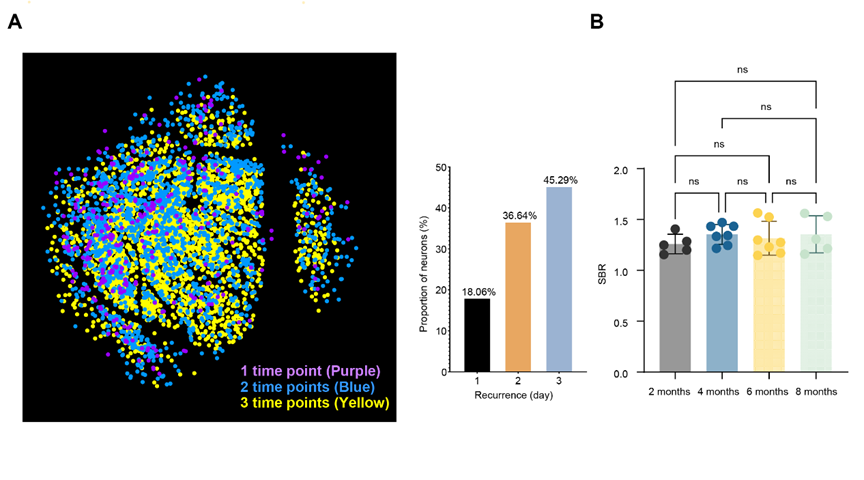


**Fig. S5. Long-term clarity of the OGPI windows.** (A) Spatially neural tracking and the bar plot of the fraction of tracked neurons across 3 time points (day 1, day 3, and day 20) in a same mouse under the same focal plane. (B) Measurements of the signal-to-background ratio (SBR) of the OGPI windows across 8 months. Data are presented as mean ± STD; N = 5, 7, 7, 5 mice; one-way ANOVA; P>0.05.

**
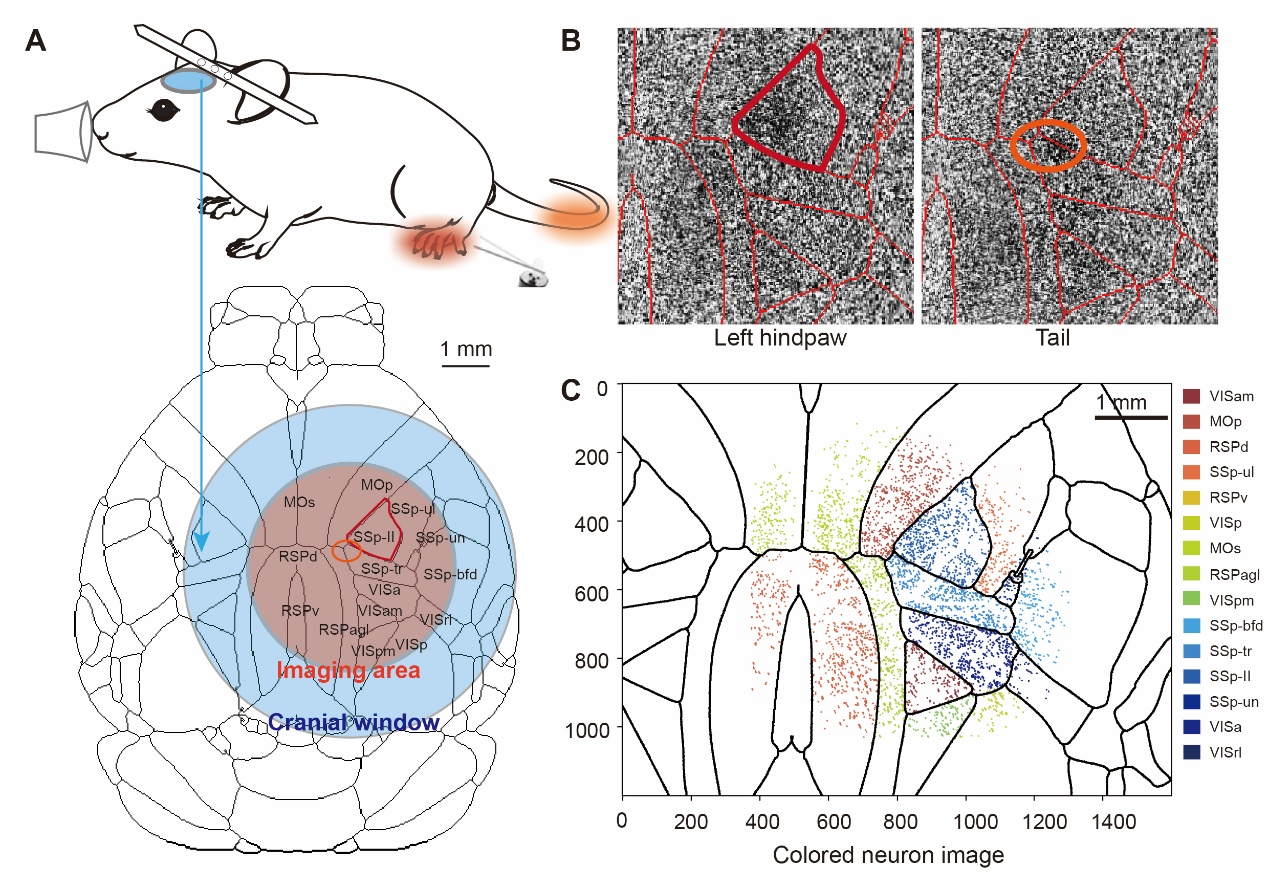
**

**Fig. S6. Intrinsic imaging to align head-mounted miniscope maps to a reference image of the cortex.** (A) A mouse with cranial window was under slight anesthesia by isoflurane (0.5-1%). We stimulated the left hind paw or the tail to get the intrinsic signal in FOV. (B) Intrinsic signals of the left hind paw (left) and the tail area (right); Bottom: we got the neuron image registration by align the intrinsic signal and midline with the Allen CCF. Abbreviations and their corresponding full names for cortical brain regions were shown in methods.


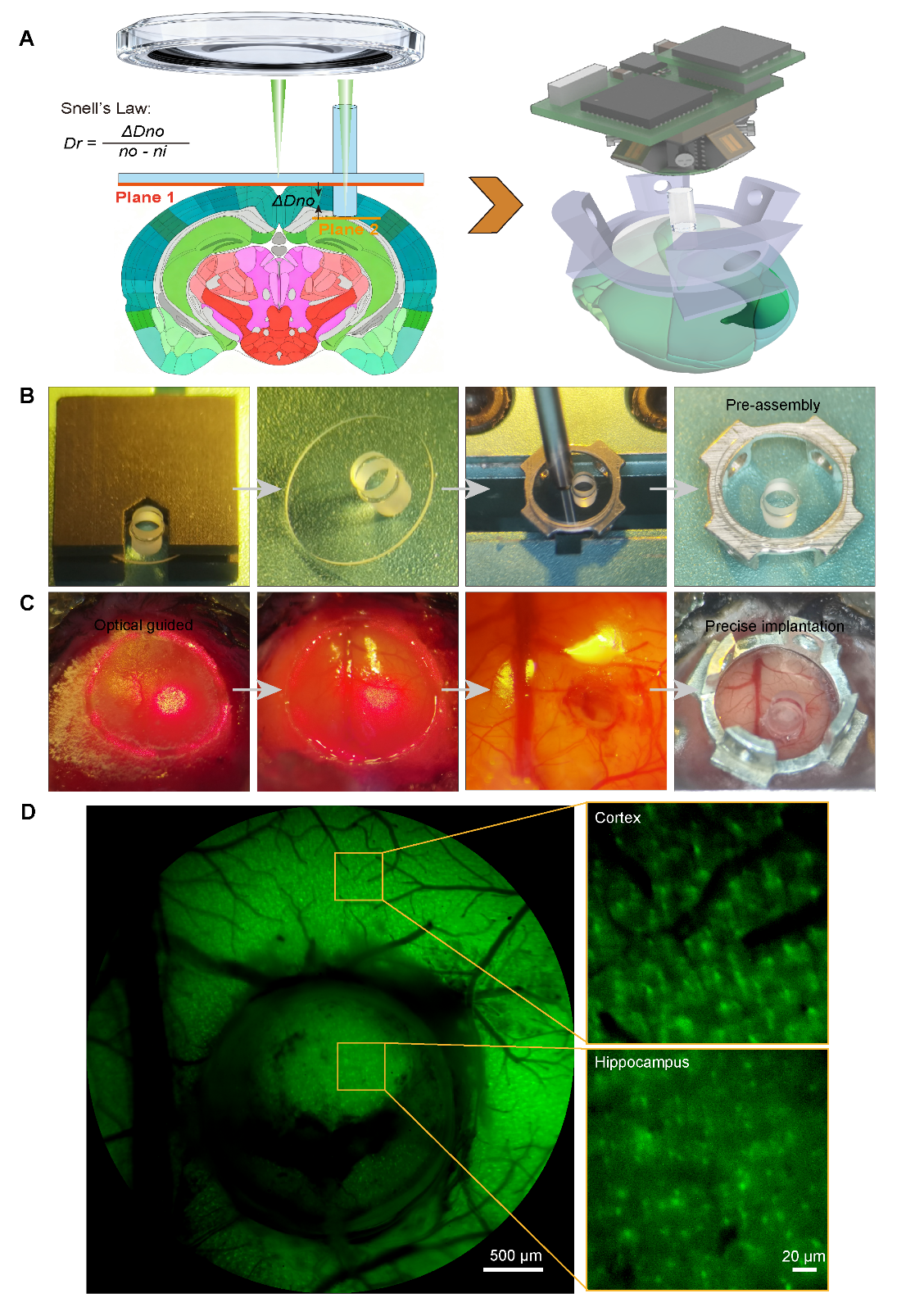


**Fig. S7. Simultaneous in vivo imaging of the cortex and the hippocampus through the OGPI cranial window.** (A) The scheme of the OGPI method for simultaneous imaging of different depths of planes. (B) Pre-assembly of the OGPI window (1 glass coverslip, two glass columns with 2 mm dia. and 0.9/1.8 mm height, and 1 metal baseplate). (C) Optical guided precise operation and implantation of the glass-metal unit. (D) Simultaneous imaging of the microglial cells in the cortex and hippocampus CA1 in a CX3CR1^GFP^ adult mouse.


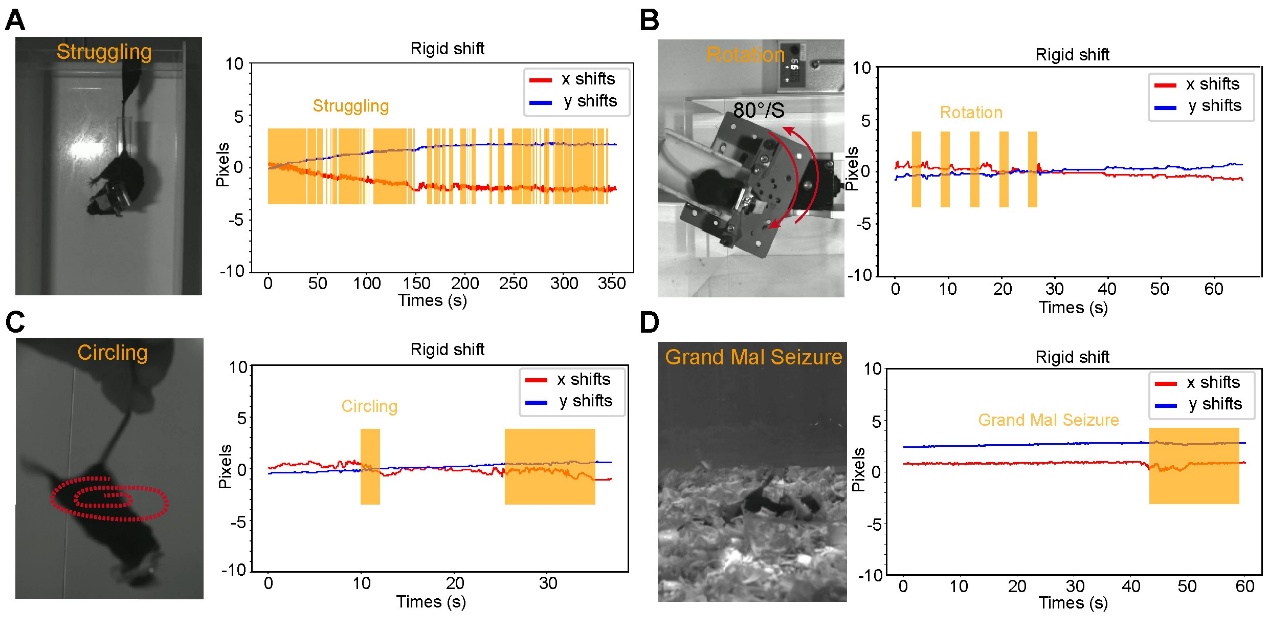


**Fig. S8. Real-time recording of rigid shift of imaging data in mice during extreme motor behaviors.** (A) Behavior and rigid shift of imaging data during the Tail Suspension Test (TST) under vigorous struggling. (B) Behavior and rigid shift of imaging data during passive rotation. (C) Circling behavior and rigid shift of imaging data in a vertigo mouse model after Unilateral Labyrinthectomy (UL). (D) Seizure behavior and rigid shift of imaging in a PTZ-induced epileptic mouse model.


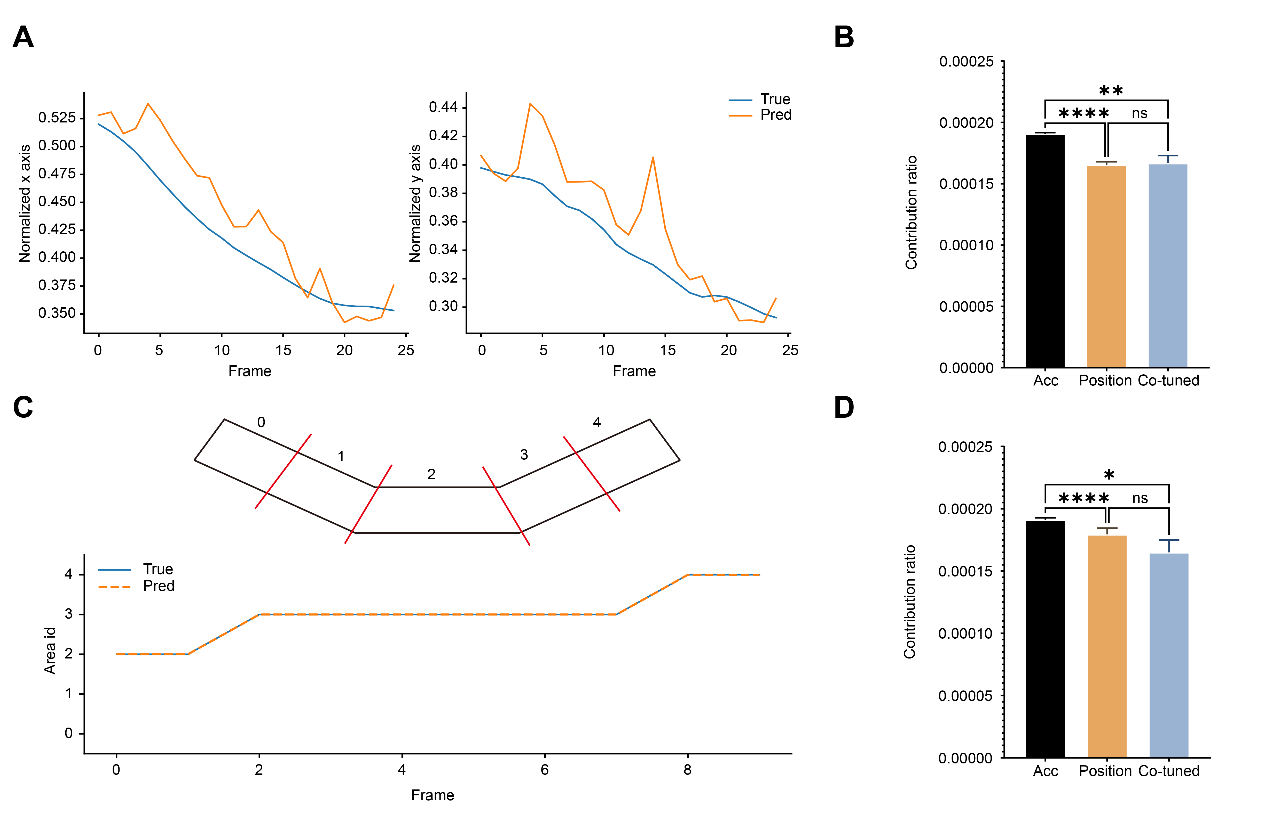


**Fig. S9. Analyze the functional significance of acceleration and spatial neurons using LSTM.** (A) Using the past 15 timepoints of neural activities as input, LSTM model predicts the trajectory of the x-axis and y-axis for the next 5 timepoints. (B) The average proportion of the backpropagated gradients of single neurons in the prediction process of three types of tuned neurons - acceleration-tuned, spatial-tuned and conjunctive-tuned. (C) Divide the y-maze into 5 areas (area id: 0-4), take the neural activities within 10 timepoints as input, and use LSTM to decode the position of the mice within these 10 timepoints. (D) The average proportion of the backpropagated gradients of single neurons in the decoding process of three types of tuned neurons - acceleration-tuned, spatial-tuned and conjunctive-tuned.


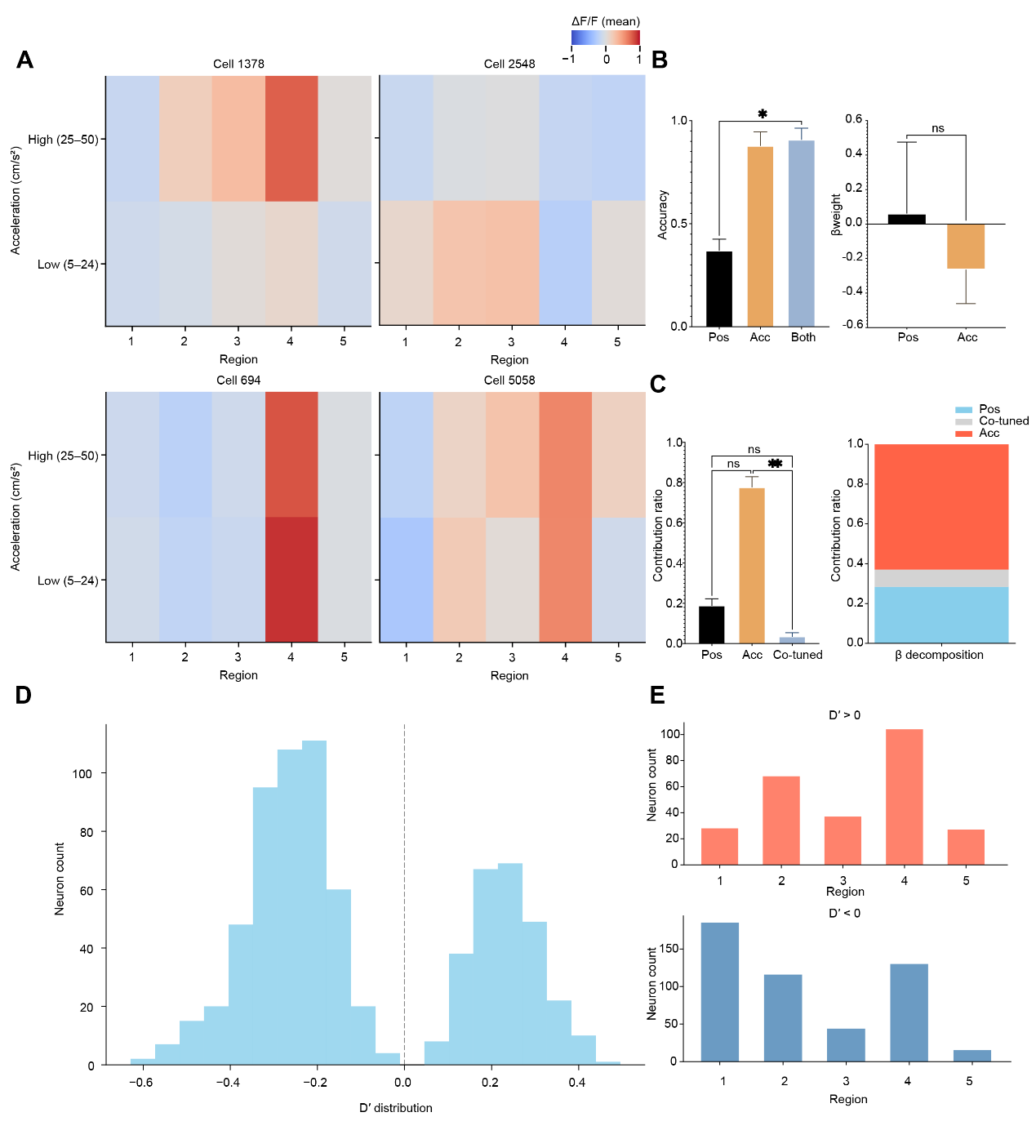


**Fig. S10. The decomposition analysis of the three types of neurons: acceleration-tuned, spatial-tuned and conjunctive -tuned.** (A) The heat maps of neural activities of the four types of neurons (high acc-tuned, low acc-tuned, spatial-tuned, conjunctive-tuned) in the Y-maze. (B) Left, the accuracy rate of fitting the X-axis trajectory using only spatial-tuned neurons, using only acceleration-tuned neurons, and using both. Right, the distribution of β weights of GLM when using both. Data are presented as mean ± STD, from 4 mice. (C)The weight contribution ratio analysis of acceleration-tuned neurons, spatial-tuned neurons, and conjunctive-tuned neurons, when using both. Data are presented as mean ± STD, from 4 mice. (D) The distribution of d' values for both high acc and low acc conditions of accelerator-tuned neurons at the same position. (E) The distribution of accelerator-tuned neurons with d' > 0 and d' < 0 at the Y-maze positions.


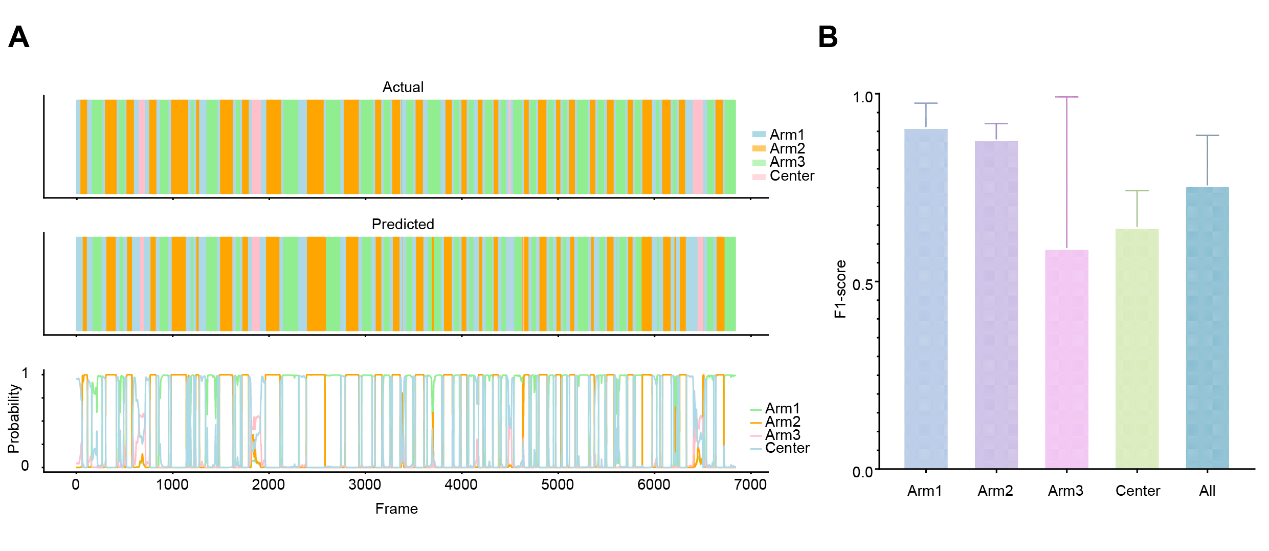


**Fig. S11. A position decoding model that solely relies on motor-related signals.** (A) Representative results of position decoding from one mouse. (B) Classifier performance metrics (F1-scores) for predicting the positions (n=4 mice). Data are presented as mean ± STD, n = 233 trials from 4 mice.


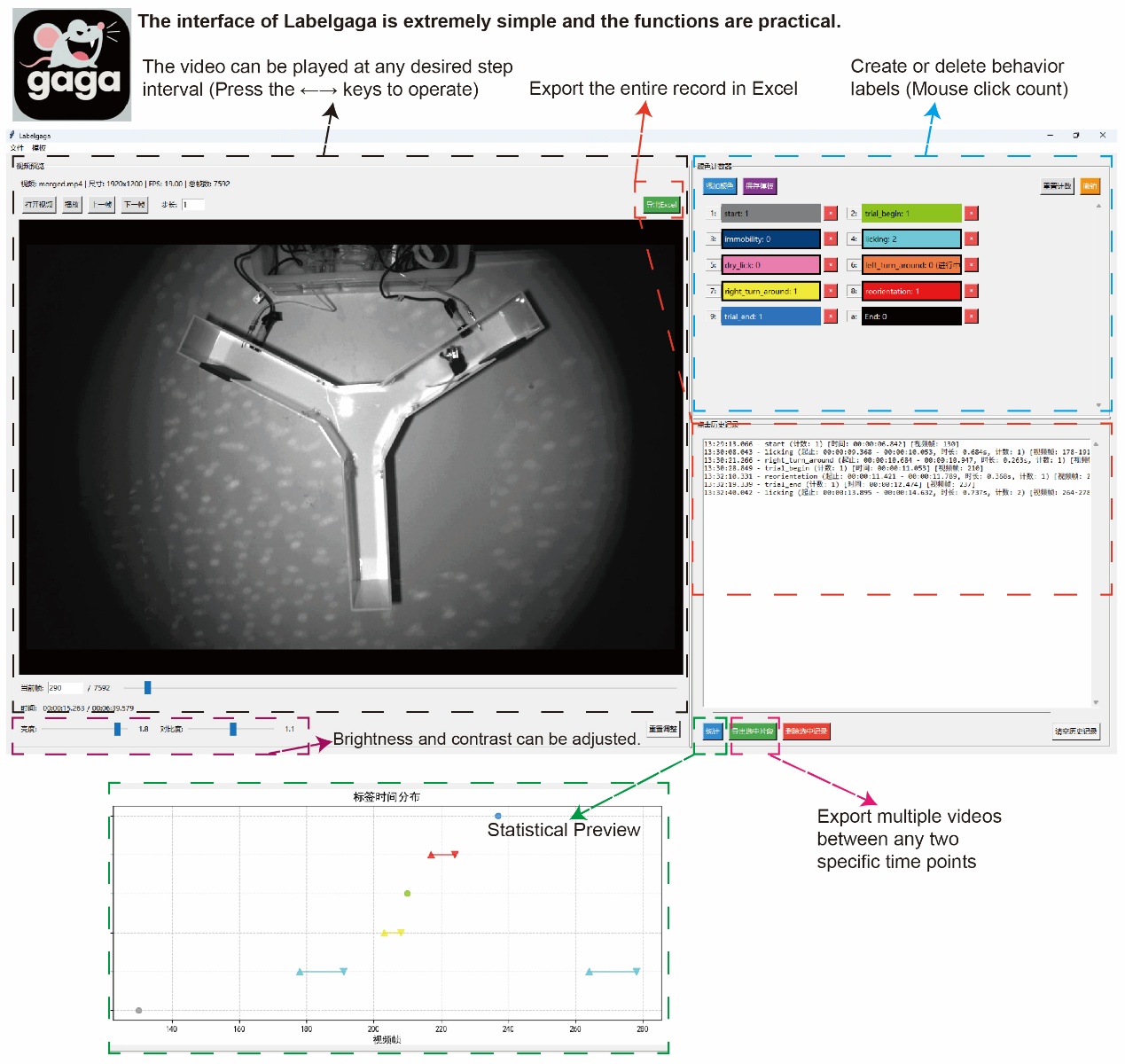


**Fig. S12. Labelgaga for annotation.** A custom-developed software for video annotation in mouse behavioral studies, featuring a smiling mouse icon. The interface is minimalist yet highly functional, allowing users to preview videos frame-by-frame using the left/right arrow keys with one hand. Simultaneously, users can adjust video brightness and contrast while using the other hand to apply predefined color-coded behavior label buttons via mouse clicking. The software supports both instantaneous event markers and continuous duration labeling. Color-coded behavior timelines can be visualized. All annotated event frames can be exported to Excel format for subsequent analysis. Users can also segment videos by clicking timeline markers as cut points, enabling export of video clips corresponding to specific behavioral epochs for detailed analysis and presentation.

**Table S1. Operation list, related to Fig. 1 and Fig. 2.**

**Table S2. A statistical summary of the OGPI method alongside other state-of-the-art techniques.**

**Movie S1. Implantation of OGPI cranial window, related to Fig. 2.**

**Movie S2. Simultaneous behavioral and neural recording before and after motion correction during TST.**

**Movie S3. Simultaneous behavioral and neural recording before and after motion correction in rotation.**

**Movie S4. Simultaneous behavioral and neural recording before and after motion correction in UL mouse model.**

**Movie S5. Simultaneous behavioral and neural recording before and after motion correction in an epileptic mouse model.**

**Movie S6. Demo of navigation trials in Y-maze, related to Fig. 4.**

REFERENCES

[1] Bahner F, Popov T, Boehme N, et al. Abstract rule learning promotes cognitive flexibility in complex environments across species [J]. Nat Commun, 2025,16(1):5396.DOI:10.1038/s41467-025-60943-7.

[2] Urai A E, Doiron B, Leifer A M, et al. Large-scale neural recordings call for new insights to link brain and behavior [J]. Nat Neurosci, 2022,25(1):11-19.DOI:10.1038/s41593-021-00980-9.

[3] Zhang Y, Wang M, Zhu Q, et al. Long-term mesoscale imaging of 3D intercellular dynamics across a mammalian organ [J]. Cell, 2024,187(21):6104-6122.DOI:10.1016/j.cell.2024.08.026.

[4] Panzeri S, Moroni M, Safaai H, et al. The structures and functions of correlations in neural population codes [J]. Nat Rev Neurosci, 2022,23(9):551-567.DOI:10.1038/s41583-022-00606-4.

[5] Shi R, Chen X, Deng J, et al. Random-access wide-field mesoscopy for centimetre-scale imaging of biodynamics with subcellular resolution [J]. Nature Photonics, 2024,18(7):721-730.DOI:10.1038/s41566-024-01422-1.

[6] Kim T H, Zhang Y, Lecoq J, et al. Long-Term Optical Access to an Estimated One Million Neurons in the Live Mouse Cortex [J]. Cell Rep, 2016,17(12):3385-3394.DOI:10.1016/j.celrep.2016.12.004.

[7] Musall S, Kaufman M T, Juavinett A L, et al. Single-trial neural dynamics are dominated by richly varied movements [J]. Nat Neurosci, 2019,22(10):1677-1686.DOI:10.1038/s41593-019-0502-4.

[8] Steinmetz N A, Aydin C, Lebedeva A, et al. Neuropixels 2.0: A miniaturized high-density probe for stable, long-term brain recordings [J]. Science, 2021,372(6539).DOI:10.1126/science.abf4588.

[9] Zhang Y, Rozsa M, Liang Y, et al. Fast and sensitive GCaMP calcium indicators for imaging neural populations [J]. Nature, 2023,615(7954):884-891.DOI:10.1038/s41586-023-05828-9.

[10] Wang S, Jiang Q, Liu H, et al. Mechanically adaptive and deployable intracortical probes enable long-term neural electrophysiological recordings [J]. Proc Natl Acad Sci U S A, 2024,121(40):e1891587175.DOI:10.1073/pnas.2403380121.

[11] Vogt N. Two-photon imaging in freely behaving mice [J]. Nat Methods, 2022,19(5):518.DOI:10.1038/s41592-022-01502-6.

[12] Zhang Y, Yuan L, Zhu Q, et al. A miniaturized mesoscope for the large-scale single-neuron-resolved imaging of neuronal activity in freely behaving mice [J]. Nat Biomed Eng, 2024,8(6):754-774.DOI:10.1038/s41551-024-01226-2.

[13] Goldey G J, Roumis D K, Glickfeld L L, et al. Removable cranial windows for long-term imaging in awake mice [J]. Nat Protoc, 2014,9(11):2515-2538.DOI:10.1038/nprot.2014.165.

[14] Takahashi T, Zhang H, Agetsuma M, et al. Large-scale cranial window for in vivo mouse brain imaging utilizing fluoropolymer nanosheet and light-curable resin [J]. Commun Biol, 2024,7(1):232.DOI:10.1038/s42003-024-05865-8.

[15] Heo C, Park H, Kim Y, et al. A soft, transparent, freely accessible cranial window for chronic imaging and electrophysiology [J]. Sci Rep, 2016,6:27818.DOI:10.1038/srep27818.

[16] Navabi Z S, Peters R, Gulner B, et al. Computer vision-guided rapid and precise automated cranial microsurgeries in mice [J]. Sci Adv, 2025,11(15):eadt9693.DOI:10.1126/sciadv.adt9693.

[17] Manita S, Shigetomi E, Bito H, et al. In Vivo Wide-Field and Two-Photon Calcium Imaging from a Mouse using a Large Cranial Window [J]. J Vis Exp, 2022(186).DOI:10.3791/64224.

[18] Augustinaite S, Kuhn B. Chronic Cranial Window for Imaging Cortical Activity in Head-Fixed Mice [J]. STAR Protoc, 2020,1(3):100194.DOI:10.1016/j.xpro.2020.100194.

[19] Hope J, Beckerle T M, Cheng P, et al. Brain-wide neural recordings in mice navigating physical spaces enabled by robotic neural recording headstages [J]. Nat Methods, 2024,21(11):2171-2181.DOI:10.1038/s41592-024-02434-z.

[20] Buzsaki G, Moser E I. Memory, navigation and theta rhythm in the hippocampal-entorhinal system [J]. Nat Neurosci, 2013,16(2):130-138.DOI:10.1038/nn.3304.

[21] Basu R, Gebauer R, Herfurth T, et al. The orbitofrontal cortex maps future navigational goals [J]. Nature, 2021,599(7885):449-452.DOI:10.1038/s41586-021-04042-9.

[22] Schneider S, Lee J H, Mathis M W. Learnable latent embeddings for joint behavioural and neural analysis [J]. Nature, 2023,617(7960):360-368.DOI:10.1038/s41586-023-06031-6.

[23] Roh H, Kim K, Kim C, et al. Polymer‐Incorporated Mechanically Compliant Carbon Nanotube Microelectrode Arrays for Multichannel Neural Signal Recording [J]. Advanced Functional Materials, 2025.DOI:10.1002/adfm.202509630.

[24] Wang Q, Ding S, Li Y, et al. The Allen Mouse Brain Common Coordinate Framework: A 3D Reference Atlas [J]. Cell, 2020,181(4):936-953.DOI:10.1016/j.cell.2020.04.007.

[25] Zong W, Obenhaus H A, Skytoen E R, et al. Large-scale two-photon calcium imaging in freely moving mice [J]. Cell, 2022,185(7):1240-1256.DOI:10.1016/j.cell.2022.02.017.

[26] Rynes M L, Ghanbari L, Schulman D S, et al. Assembly and operation of an open-source, computer numerical controlled (CNC) robot for performing cranial microsurgical procedures [J]. Nat Protoc, 2020,15(6):1992-2023.DOI:10.1038/s41596-020-0318-4.

[27] Padmashri R, Tyner K, Dunaevsky A. Implantation of a Cranial Window for Repeated In Vivo Imaging in Awake Mice [J]. J Vis Exp, 2021(172).DOI:10.3791/62633.

[28] Oomoto I, Uwamori H, Matsubara C, et al. Protocol for cortical-wide field-of-view two-photon imaging with quick neonatal adeno-associated virus injection [J]. STAR Protoc, 2021,2(4):101007.DOI:10.1016/j.xpro.2021.101007.

[29] Zhong J, Gunner G, Henninger N, et al. Intravital Imaging of Fluorescent Protein Expression in Mice with a Closed-Skull Traumatic Brain Injury and Cranial Window Using a Two-Photon Microscope [J]. J Vis Exp, 2023(194).DOI:10.3791/64701.

[30] Kyweriga M, Sun J, Wang S, et al. A Large Lateral Craniotomy Procedure for Mesoscale Wide-field Optical Imaging of Brain Activity [J]. J Vis Exp, 2017(123).DOI:10.3791/52642.

[31] Huisman T A G M, Tschirch F T C. Epidural hematoma in children: do cranial sutures act as a barrier? [J]. J Neuroradiol, 2009,36(2):93-97.DOI:10.1016/j.neurad.2008.06.003.

[32] Li B, Li J, Fan Y, et al. Dissecting calvarial bones and sutures at single-cell resolution [J]. Biol Rev Camb Philos Soc, 2023,98(5):1749-1767.DOI:10.1111/brv.12975.

[33] Jovanovic N, Suchankova S, Kang M, et al. Altered hearing function in mice with implanted cranial windows [J]. Neurosci Lett, 2023,792:136969.DOI:10.1016/j.neulet.2022.136969.

[34] Li H, Spitzer N C. Exercise enhances motor skill learning by neurotransmitter switching in the adult midbrain [J]. Nat Commun, 2020,11(1):2195.DOI:10.1038/s41467-020-16053-7.

[35] Campagner D, Vale R, Tan Y L, et al. A cortico-collicular circuit for orienting to shelter during escape [J]. Nature, 2023,613(7942):111-119.DOI:10.1038/s41586-022-05553-9.

[36] Deitch D, Rubin A, Ziv Y. Representational drift in the mouse visual cortex [J]. Curr Biol, 2021,31(19):4327-4339.DOI:10.1016/j.cub.2021.07.062.

[37] Moser E I, Moser M, McNaughton B L. Spatial representation in the hippocampal formation: a history [J]. Nat Neurosci, 2017,20(11):1448-1464.DOI:10.1038/nn.4653.

[38] Bowler J C, Losonczy A. Direct cortical inputs to hippocampal area CA1 transmit complementary signals for goal-directed navigation [J]. Neuron, 2023,111(24):4071-4085.DOI:10.1016/j.neuron.2023.09.013.

[39] Issa J B, Radvansky B A, Xuan F, et al. Lateral entorhinal cortex subpopulations represent experiential epochs surrounding reward [J]. Nat Neurosci, 2024,27(3):536-546.DOI:10.1038/s41593-023-01557-4.

[40] Keshavarzi S, Bracey E F, Faville R A, et al. Multisensory coding of angular head velocity in the retrosplenial cortex [J]. Neuron, 2022,110(3):532-543.DOI:10.1016/j.neuron.2021.10.031.

[41] Hennestad E, Witoelar A, Chambers A R, et al. Mapping vestibular and visual contributions to angular head velocity tuning in the cortex [J]. Cell Rep, 2021,37(12):110134.DOI:10.1016/j.celrep.2021.110134.

[42] Long X, Zhang S. A novel somatosensory spatial navigation system outside the hippocampal formation [J]. Cell Res, 2021,31(6):649-663.DOI:10.1038/s41422-020-00448-8.

[43] Fiser A, Mahringer D, Oyibo H K, et al. Experience-dependent spatial expectations in mouse visual cortex [J]. Nat Neurosci, 2016,19(12):1658-1664.DOI:10.1038/nn.4385.

[44] Noel J, Angelaki D E. Cognitive, Systems, and Computational Neurosciences of the Self in Motion [J]. Annu Rev Psychol, 2022,73:103-129.DOI:10.1146/annurev-psych-021021-103038.

[45] Harvey R E, Rutan S A, Willey G R, et al. Linear Self-Motion Cues Support the Spatial Distribution and Stability of Hippocampal Place Cells [J]. Curr Biol, 2018,28(11):1803-1810.DOI:10.1016/j.cub.2018.04.034.

[46] Valerio S, Taube J S. Head Direction Cell Activity Is Absent in Mice without the Horizontal Semicircular Canals [J]. J Neurosci, 2016,36(3):741-754.DOI:10.1523/JNEUROSCI.3790-14.2016.

[47] Arvaniti C K, Brotis A G, Paschalis T, et al. Localization of Vestibular Cortex Using Electrical Cortical Stimulation: A Systematic Literature Review [J]. Brain Sci, 2024,14(1).DOI:10.3390/brainsci14010075.

[48] Zhou Z, Tian E, Wang J, et al. Cognitive impairments and neurobiological changes induced by unilateral vestibular dysfunction in mice [J]. Neurobiol Dis, 2024,202:106719.DOI:10.1016/j.nbd.2024.106719.

[49] Barbour A J, Gourmaud S, Lancaster E, et al. Seizures exacerbate excitatory: inhibitory imbalance in Alzheimer's disease and 5XFAD mice [J]. Brain, 2024,147(6):2169-2184.DOI:10.1093/brain/awae126.

[50] Jacob A D, Ramsaran A I, Mocle A J, et al. A Compact Head-Mounted Endoscope for In Vivo Calcium Imaging in Freely Behaving Mice [J]. Curr Protoc Neurosci, 2018,84(1):e51.DOI:10.1002/cpns.51.

[51] Shen H, Zhang C, Zhang Q, et al. Gut microbiota modulates depressive-like behaviors induced by chronic ethanol exposure through short-chain fatty acids [J]. J Neuroinflammation, 2024,21(1):290.DOI:10.1186/s12974-024-03282-6.

[52] Sclafani A, Ackroff K. Operant licking for intragastric sugar infusions: Differential reinforcing actions of glucose, sucrose and fructose in mice [J]. Physiol Behav, 2016,153:115-124.DOI:10.1016/j.physbeh.2015.10.021.

[53] Gonzalo Cogno S, Obenhaus H A, Lautrup A, et al. Minute-scale oscillatory sequences in medial entorhinal cortex [J]. Nature, 2024,625(7994):338-344.DOI:10.1038/s41586-023-06864-1.

[54] Steiner A P, Redish A D. The road not taken: neural correlates of decision making in orbitofrontal cortex [J]. Front Neurosci, 2012,6:131.DOI:10.3389/fnins.2012.00131.

[55] Ravi N, Gabeur V, Hu Y, et al. SAM 2: Segment Anything in Images and Videos [J]. ArXiv, 2024,abs/2408.00714.

[56] Ueno H, Takahashi Y, Murakami S, et al. Effect of simultaneous testing of two mice in the tail suspension test and forced swim test [J]. Sci Rep, 2022,12(1):9224.DOI:10.1038/s41598-022-12986-9.

[57] Yuanlong Z, Lekang Y, Mingrui W, et al. An ultracompact wireless mesoscope for large-scale neural recording during fully unconstrained behavior [C]//: Proc.SPIE, 2025.2025-03-21.

[58] Pnevmatikakis E A, Giovannucci A. NoRMCorre: An online algorithm for piecewise rigid motion correction of calcium imaging data [J]. J Neurosci Methods, 2017,291:83-94.DOI:10.1016/j.jneumeth.2017.07.031.

[59] Zhang Y, Zhang G, Han X, et al. Rapid detection of neurons in widefield calcium imaging datasets after training with synthetic data [J]. Nat Methods, 2023,20(5):747-754.DOI:10.1038/s41592-023-01838-7.

[60] Hochreiter S, Schmidhuber J. Long short-term memory [J]. Neural Comput, 1997,9(8):1735-1780.DOI:10.1162/neco.1997.9.8.1735.

[61] Ebrahimi S, Lecoq J, Rumyantsev O, et al. Emergent reliability in sensory cortical coding and inter-area communication [J]. Nature, 2022,605(7911):713-721.DOI:10.1038/s41586-022-04724-y.

[62] Xie H, Han X, Xiao G, et al. Multifocal fluorescence video-rate imaging of centimetre-wide arbitrarily shaped brain surfaces at micrometric resolution [J]. Nat Biomed Eng, 2024,8(6):740-753.DOI:10.1038/s41551-023-01155-6.

[63] Fan J, Suo J, Wu J, et al. Video-rate imaging of biological dynamics at centimetre scale and micrometre resolution [J]. Nature Photonics, 2019,13(11):809-816.DOI:10.1038/s41566-019-0474-7.

[64] Ghanbari L, Carter R E, Rynes M L, et al. Cortex-wide neural interfacing via transparent polymer skulls [J]. Nat Commun, 2019,10(1):1500.DOI:10.1038/s41467-019-09488-0.

[65] Rynes M L, Surinach D A, Linn S, et al. Miniaturized head-mounted microscope for whole-cortex mesoscale imaging in freely behaving mice [J]. Nat Methods, 2021,18(4):417-425.DOI:10.1038/s41592-021-01104-8.

[66] Padmashri R, Tyner K, Dunaevsky A. Implantation of a Cranial Window for Repeated In Vivo Imaging in Awake Mice [J]. J Vis Exp, 2021(172).DOI:10.3791/62633.

[67] Zhang R, Zhuang C, Wang Z, et al. Simultaneous Observation of Mouse Cortical and Hippocampal Neural Dynamics under Anesthesia through a Cranial Microprism Window [J]. Biosensors (Basel), 2022,12(8).DOI:10.3390/bios12080567.

[68] Edelman B J, Siegenthaler D, Wanken P, et al. The COMBO window: A chronic cranial implant for multiscale circuit interrogation in mice [J]. PLoS Biol, 2024,22(6):e3002664.DOI:10.1371/journal.pbio.3002664.

[69] Tournissac M, Boido D, Omnes M, et al. Cranial window for longitudinal and multimodal imaging of the whole mouse cortex [J]. Neurophotonics, 2022,9(3):31921.DOI:10.1117/1.NPh.9.3.031921.

[70] Kilic K, Desjardins M, Tang J, et al. Chronic Cranial Windows for Long Term Multimodal Neurovascular Imaging in Mice [J]. Front Physiol, 2020,11:612678.DOI:10.3389/fphys.2020.612678.

[71] Yang N, Liu F, Zhang X, et al. A Hybrid Titanium-Softmaterial, High-Strength, Transparent Cranial Window for Transcranial Injection and Neuroimaging [J]. Biosensors (Basel), 2022,12(2).DOI:10.3390/bios12020129.

[72] Mikkelsen S H, Wied B, Dashkovskyi V, et al. Head holder and cranial window design for sequential magnetic resonance imaging and optical imaging in awake mice [J]. Front Neurosci, 2022,16:926828.DOI:10.3389/fnins.2022.926828.

[73] Yang Q, Vazquez A L, Cui X T. Long-term in vivo two-photon imaging of the neuroinflammatory response to intracortical implants and micro-vessel disruptions in awake mice [J]. Biomaterials, 2021,276:121060.DOI:10.1016/j.biomaterials.2021.121060.

[74] Yin R, Noble B C, He F, et al. Chronic co-implantation of ultraflexible neural electrodes and a cranial window [J]. Neurophotonics, 2022,9(3):32204.DOI:10.1117/1.NPh.9.3.032204.

[75] Kim J U, Park H, Ok J, et al. Cerebrospinal Fluid-philic and Biocompatibility-Enhanced Soft Cranial Window for Long-Term In Vivo Brain Imaging [J]. ACS Appl Mater Interfaces, 2022,14(13):15035-15046.DOI:10.1021/acsami.2c01929.

[76] Zuluaga-Ramirez V, Rom S, Persidsky Y. Craniula: A cranial window technique for prolonged imaging of brain surface vasculature with simultaneous adjacent intracerebral injection [J]. Fluids Barriers CNS, 2015,12:24.DOI:10.1186/s12987-015-0021-y.

[77] Zong W, Wu R, Li M, et al. Fast high-resolution miniature two-photon microscopy for brain imaging in freely behaving mice [J]. Nat Methods, 2017,14(7):713-719.DOI:10.1038/nmeth.4305.

[78] Guo C, Blair G J, Sehgal M, et al. Miniscope-LFOV: A large-field-of-view, single-cell-resolution, miniature microscope for wired and wire-free imaging of neural dynamics in freely behaving animals [J]. Sci Adv, 2023,9(16):eadg3918.DOI:10.1126/sciadv.adg39
